# *In silico* predicted therapy against chronic *Staphylococcus aureus* infection leads to bacterial clearance *in vivo*

**DOI:** 10.1016/j.isci.2022.105522

**Published:** 2022-11-08

**Authors:** Lito A. Papaxenopoulou, Gang Zhao, Sahamoddin Khailaie, Konstantinos Katsoulis-Dimitriou, Ingo Schmitz, Eva Medina, Haralampos Hatzikirou, Michael Meyer-Hermann

**Affiliations:** 1 Department of Systems Immunology and Braunschweig Integrated Centre of Systems Biology, Helmholtz Centre for Infection Research, 38106 Braunschweig, Germany; 2 Faculty of Life Sciences, Technical University Braunschweig, 38106 Braunschweig, Germany; 3 Institute for Molecular and Clinical Immunology, Medical Faculty, Otto-von-Guericke-University, 39120 Magdeburg, Germany; 4 Systems-Oriented Immunology and Inflammation Research Group, Department of Experimental Immunology, Helmholtz Centre for Infection Research, 38124 Braunschweig, Germany; 5 Department of Molecular Immunology, ZKF2, Medical Faculty, Ruhr-University Bochum, 44780 Bochum, Germany; 6 Department of Infection Immunology, Helmholtz Centre for Infection Research, 38124 Braunschweig, Germany; 7 Technische Univesität Dresden, Center for Information Services and High Performance Computing, 01062 Dresden, Germany; 8 Mathematics Department, Khalifa University, 127788 Abu Dhabi, UAE; 9 Institute for Biochemistry, Biotechnology and Bioinformatics, Technische Universität Braunschweig, 38106 Braunschweig, Germany

**Keywords:** Immunity

## Abstract

*Staphylococcus aureus* can lead to chronic infections and abscesses in internal organs including kidneys, which are associated with the expansion of myeloid-derived suppressor cells (MDSCs) and their suppressive effect on T cells. Here, we developed a mathematical model of chronic *S. aureus* infection that incorporates the T-cell suppression by MDSCs and suggests therapeutic strategies for *S. aureus* clearance. A therapeutic protocol with heat-killed *S. aureus* (HKSA) was quantified *in silico* and tested *in vivo*. Contrary to the conventional administration of heat-killed bacteria as vaccination prior to infection, we administered HKSA as treatment in chronically infected hosts. Our treatment eliminated *S. aureus* in kidneys of all chronically *S. aureus*-infected mice, reduced MDSCs, and reversed T-cell dysfunction by inducing acute inflammation during ongoing, chronic infection. This study is a guideline for a treatment protocol against chronic *S. aureus* infection and renal abscesses by repurposing heat-killed treatments, directed by mathematical modeling.

## Highlights

Model-driven quantified therapy protocol design against chronic *Staphylococcus aureus* infection
• Treatment with heat-killed *S. aureus* eradicates *S. aureus in vivo*
• Treatment induces acute inflammation during ongoing, chronic *S. aureus* infection
• A proof-of-principle against renal *S. aureus* abscesses

## Graphical abstract

**Figure:**
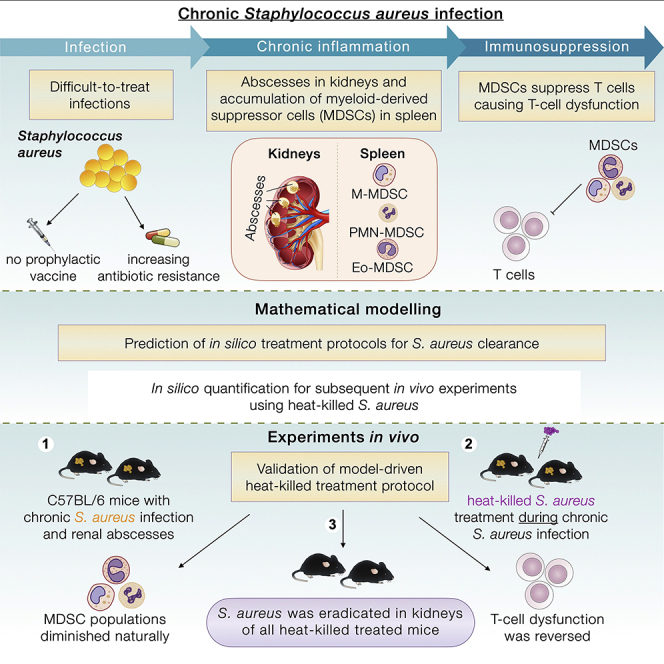

## Introduction

*S. aureus* is a bacterial human pathogen colonizing 20-30% of the world population and is responsible for nosocomial-acquired and community-acquired infections. *S. aureus* can colonize asymptomatically the human skin as a commensal bacterium. However, after a skin cut, surgery, or implantation of medical devices, *S. aureus* can reach deeper tissues and can cause life-threatening conditions like pneumonia, endocarditis, osteomyelitis, and abscesses in internal organs.^1^^,^^2^

The pathogen is of substantial medical concern because it can infect any organ in the body,^1^^,^^2^ even without being disseminated via the bloodstream.^3^ Moreover, its multiple mechanisms to manipulate and evade immune defenses along with its increasing antibiotic-resistance lead to its persistence in the host and cause chronic, difficult-to-treat infections.^4^^,^^5^ The urgency for new treatments aiming at curing *S. aureus* infections is also emphasized by the WHO, which, on its global priority list of infectious agents, identified *S. aureus* as a “high-priority” pathogen.

Chronic *S. aureus* infections such as chronic osteomyelitis,^6^ recurrent furunculosis,^7^ and abscesses^8^^,^^9^ are hard to eliminate. We have previously shown that during the *chronic phase* of *S. aureus* infection, effectors of innate immunity, such as macrophages (MΦ) and neutrophils, as well as B cells are dispensable for bacterial containment, unlike T cells, which are critical for bacterial control.^10^ However, T cells fail to eradicate the pathogen because prolonged antigenic stimulation (confluent with chronic infection) causes them to enter an anergic state that seems to be irreversible.^10^ As we demonstrated, T-cell dysfunction (also known as T-cell anergy, T-cell suppression, or T-cell hyporesponsiveness) during chronic *S. aureus* infection is attributed to myeloid-derived suppressor cells (MDSCs) rather than other immunosuppressive cells such as regulatory T and B cells, or tolerogenic dendritic cells.^9^

MDSCs constitute a heterogeneous population of immature myeloid cells that expand in long-lasting pathological conditions, such as chronic bacterial and viral infections^11^^,^^12^ including severe SARS-CoV-2 infection,^13^ cancer,^14^ and autoimmunity.^15^ Expansion of MDSCs serves as a natural, anti-inflammatory response to mitigate the detrimental effect of prolonged inflammation.^5^ Often pathogens exploit the immunosuppressive effect by MDSCs to persist within the host, thus establishing chronic infections.^5^ MDSCs are distinguished in three subsets that share the capacity to suppress T cells during chronic *S. aureus* infection: monocytic CD11b^+^Ly6C^+^Ly6G^low^, granulocytic/neutrophilic CD11b^+^Ly6C^low^Ly6G^+^ and eosinophilic CD11b^+^Ly6C^low^Ly6G^low^ MDSCs, known as M-MDSC, PMN-MDSC and Eo-MDSC respectively.^9^^,^^16^

Despite much research, there is no *S. aureus* vaccine to confer prophylaxis against *S. aureus* infections. In 2014, 616,070 US inpatients were afflicted with methicillin-susceptible and methicillin-resistant *S. aureus* alone, and associated costs were estimated to be around $14.6 billion.^17^

A common manifestation of *S. aureus* infections, including those by antibiotic-resistant strains, is the formation of abscesses.^8^^,^^18^^,^^19^^,^^20^ Current treatments rely on the simultaneous use of various antibiotics (which promote antibiotic resistance), MDSC-targeting drugs (which are cytotoxic and can have side effects), and surgery to drain abscesses. Renal abscesses in particular are highly destructive and when multiple antibiotics and abscess drainage fail, immediate nephrectomy is required to save a patient’s life.^21^ Consequently, finding new treatments against *S. aureus* infections is absolutely essential.

Experimental investigations have offered important information on mechanisms of bacterial persistence or MDSC-mediated immunosuppression, however, how to intervene in the complex balance between bacteria, T cells, and MDSCs during chronic *S. aureus* infections in order to resolve the infection, remains obscure. In this study, we constructed a mathematical model to understand the balance between immunity and *S. aureus* during chronic infection and to explore strategies that could clear the infection. Modeling the chronic infection mathematically could bestow a broader observation of possible treatments that would be challenging to discover only by experimental means, whereas *in silico* they could expeditiously and cost-effectively be tested for rendering bacterial clearance. Mathematical models have been used to shed light on *S. aureus* transmission in community and hospitals,^22^^,^^23^ staphylococcal growth on foods,^24^ interactions of the pathogen and immune cells during the acute phase of infection,^25^ but also to suggest the optimal sequence of antibiotic administration that could reduce the virulence of *S. aureus*.^26^ However, there are still large gaps on chronic *S. aureus* infections and how to resolve them. To our knowledge, this is the first mathematical model that investigates the dynamics of *chronic S. aureus* infection between bacteria and T cells in the presence of MDSCs, and suggests therapeutic treatments.

Our *in silico* analysis suggested various strategies that could perturb the dynamics of the chronic infection system and clear the infection. For experimental testing *in vivo*, we quantified *in silico* a dose-day treatment protocol using heat-killed (HK), namely inactivated, bacteria. Unlike prior vaccination with HK *S. aureus* (HKSA), which is meant as prophylaxis but instead fails to eradicate the pathogen and exacerbates the infection,^27^ we administered HKSA as treatment, when the hosts were already chronically *S. aureus-*infected. Our *in silico* therapeutic protocol was validated *in vivo*. We here report for the first time reversion of T-cell dysfunction, MDSC-reduction, and eradication of *S. aureus* in the kidneys of all HKSA-treated mice without any use of antibiotics, MDSC-targeting drugs, or procedures such as abscess drainage. Our experiments further verified that our HKSA protocol triggered acute inflammation during the already established chronic *S. aureus* infection, which served as the perturbation of the system dynamics and cleared the infection. The therapeutic effect of heat-killed treatment is not limited to HKSA, because treatment with heat-killed *Streptococcus pyogenes* (HKSP) also led to bacterial clearance in a portion of treated animals, reverted T-cell dysfunction, and induced acute inflammation during ongoing chronic *S. aureus* infection. Our study is a proof-of-principle for a treatment protocol against chronic *S. aureus* infection and renal abscesses by repurposing heat-killed administration, guided and quantified by mathematical modeling that may have direct relevance to the design of human therapeutics against chronic *S. aureus* infections and abscesses in internal organs.

## Results

### Dynamics of chronic infection

We have previously shown that intravenous inoculation with *S. aureus* strain SH1000 in C57BL/6 mice results in chronic infection and abscesses in kidneys.^9^^,^^10^ Bacterial containment in the chronic phase of *S. aureus* SH1000 infection is not attributed to innate immune cells but mainly to CD4^+^ T cells, which gradually lose functionality due to suppression by MDSCs.^9^^,^^10^ Here, we constructed a mathematical model that includes the above-mentioned interactions between bacteria *B(t)*, T cells*T(t)*, and T-cell suppression by MDSCs (parameter Θ) during *chronic S. aureus* infection. In the following the term T cells will refer to CD4^+^ T cells unless otherwise stated.

The ordinary differential equation (ODE) system reads

(Equation 1) $$\dot{B}\left(t\right)=r_{b}B\left(t\right)\left(1-\frac{B\left(t\right)}{\kappa }\right)-c_{b}T\left(t\right)B\left(t\right)\text{,}$$

(Equation 2) $$\dot{T}\left(t\right)=r_{t}T^{2}\left(t\right)\left(1-\frac{T\left(t\right)}{K_{T}}\right)+k_{b}B\left(t\right)-c_{T}B\left(t\right)T\left(t\right)-\text{Θ}\;T\left(t\right)\text{,}$$

where a dot represents differentiation with respect to time.

**Figure 1:**
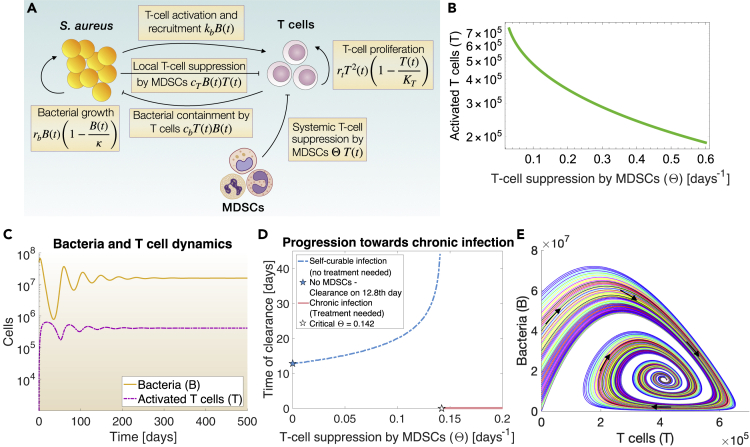
Dynamics of chronic *Staphylococcus aureus* infection (A) Schematic representation of chronic *S. aureus* infection model. (B) Correlation between T cells and T-cell suppression by MDSCs (Θ) is inversely proportional. The correlation was plotted using the analytical solutions of the T-cell differential equation in steady state (STAR Methods, Equation S5) for increasing values of parameter Θ. (C) Dynamics of bacteria (B) and activated T cells (T) in time are shown as numerical solutions of the ODE system. Infection was induced by setting the bacterial population equal to 5×10^7^ cells at day 0 in Equation 1 to be consistent with experimental bacterial inoculation causing chronic *S. aureus* infection.^9^ Model parameters are shown in Table S1. (D) Infection was initiated as in (C). Numerical calculation of bacteria for changing values of parameter Θ (T-cell suppression by MDSCs) in the range [0, 0.2]. For bacterial numbers <0.000001, the infection is considered resolved (blue), else persisting (pink) and the corresponding day of bacterial clearance is shown or set to zero, respectively. The blue star represents a scenario of MDSC-absence and hence non-existent T-cell suppression (Θ = 0). As Θ gradually increases, the infection progress toward the chronic phase and bacterial clearance becomes more difficult. The blue line represents when, despite MDSC-mediated suppression, the infection can be cleared by the immune response alone. The white star represents the critical value of Θ, when T cells become anergic by MDSC-mediated suppression, the infection persists and external treatment intervention is required for bacterial clearance. Table S1 shows the values for the rest of the model parameters. (E) Stable steady state of ODE system between bacteria and T cells. For changing initial numbers of bacteria in the range [10^5^, 6 × 10^7^) at day 0, the system always terminates in stable equilibrium, which biologically corresponds to the chronic infection. Black arrows illustrate the flow of the system. Model parameters are shown in Table S1.

During infection, *S. aureus* uses various mechanisms to persist within the host.^28^ This is represented by the term *r*_*b*_*B*(*t*)(1−*B*(*t*)/*κ*) capturing bacterial expansion by logistic growth. Staphylococcal presence stimulates T cells (term *k*_*b*_*B*(*t*)), which proliferate (term *r*_*t*_*T*^2^(*t*)). T-cell proliferation is represented with the term *r*_*t*_*T*^2^(*t*) because activated T cells secrete Interleukin-2 (IL-2), which induces cell cycle progression of T cells.^29^ In return, it creates a positive feedback loop for T-cell proliferation,^29^ and hence the term *r*_*t*_*T*^2^(*t*) as we reported previously.^30^ The term (1−*T*(*t*)/*K*_*T*_) describes the carrying capacity of T cells. As infection becomes chronic, T cells contain bacteria (term *c*_*b*_*T*(*t*)*B*(*t*)).^10^ However, bacterial persistence causes chronic (long-lasting) inflammation, which is harmful to the host. To protect from the adverse effect of prolonged inflammatory signal, MDSCs gradually expand to suppress T-cell activity.^9^ T-cell suppression by MDSCs can happen *systemically,* namely distantly from the site of infection such as in the spleen,^9^^,^^16^ where T cells increase substantially during chronic *S. aureus* infection.^10^ This is represented by the term Θ *T*(*t*), since MDSC-mediated immunosuppression on T cells requires direct cell-cell contact or cell-cell proximity.^9^ At the site of infection, *S. aureus B(t)* is able to induce a *local* immunosuppressive microenvironment that suppresses T cells to promote its persistence (term *c*_*T*_*B*(*t*)*T*(*t*)) via various immune evasion strategies for instance, through the induction of interleukins IL-10 and IL-27, or *S. aureus* enterotoxins, which promote the expansion and functions of MDSCs leading to T-cell suppression.^4^^,^^5^^,^^31^^,^^32^ A schematic representation of the model is illustrated in Figure 1A. It is important to note that although innate immune responses do not explicitly appear in the equations, they were not ignored but were rather indirectly incorporated into the parameters describing bacterial growth, *r*_*b*_ and *κ,* antigen-presenting cell (APC) activity via the parameter *k*_*b*_ and the steady-state activity of phagocytes via the parameter *c*_*b*_. In particular, model parameters were identified with the use of our previously reported experimental results from T and B cells-deficient RAG2^−/−^ mice (only innate immunity present) and immunocompetent mice with chronic *S. aureus* infection and renal abscesses^10^ (full description in STAR Methods). We previously reported major T-cell suppression by MDSCs in spleens of mice with local, chronic *S. aureus* infection in kidneys.^9^^,^^16^ This phenomenon is known as extramedullar hematopoiesis, happens during chronic inflammation and involves hematopoiesis mostly in spleen, which further induces the accumulation of MDSCs in the organ.^33^ In accordance, the fitted value for parameter *c*_*T*_ multiplied by *B(t)* and *T(t)* in the term *c*_*T*_*B*(*t*)*T*(*t*) describing local T-cell suppression in kidneys (the site of infection) was much smaller compared to parameter Θ multiplied by *T(t)* in the term Θ *T*(*t*), describing systemic T-cell suppression, such as in spleen (details in STAR Methods).

We previously demonstrated experimentally that during chronic *S. aureus* infection there is the substantial negative correlation between MDSC populations and activated T cells, which are not suppressed (not dysfunctional) and hence maintain their ability to proliferate.^9^ To validate the accuracy and consistency of our mathematical model, we reproduced the inverse proportional behavior between T cells and T-cell suppression by MDSCs (Figure 1B). Further *in silico* analysis showed how chronic *S. aureus* infection is established naturally, without any treatment intervention (Figure 1C). Infection induces strong inflammation, which activates T cells Competition for dominance between bacteria and T cells creates oscillations in the population dynamics (Figure 1C). To protect from the damaging effect of prolonged inflammation, MDSCs expand gradually to suppress T cells. Increasing accumulation of MDSCs leads to increasing suppression on T cells (Θ). Upon a critical threshold, T-cell suppression by MDSCs is so strong that T cells become dysfunctional and cannot promote bacterial clearance anymore (Figure 1D, *pink line*). This *in silico* result agrees with our previous experimental observations, showing that gradual expansion of MDSCs leads to gradual loss of T-cell function, which in turn promotes chronic *S. aureus* infection and failure of bacterial clearance.^9^^,^^10^ At this point the immune system finds the balance between maximal bacterial clearance and minimal collateral tissue damage, whereas bacteria persist in the host organism but are simultaneously unable to further grow due to their containment by T cells^10^ (mathematically known as the equilibrium of the system). This equilibrium is found to be stable by our mathematical model (Figure 1E) and biologically refers to chronic *S. aureus* infection. Once at this stage (Figure 1D, *pink line;* 1E), bacterial clearance can be attained only by using treatment interventions that can destabilize (i.e. perturb) this stable steady state of chronic infection. For all *in silico* results, the day of clearance was defined as the first time-point when bacterial numbers were <0.000001.

### Model-driven therapeutic strategies

**Figure 2:**
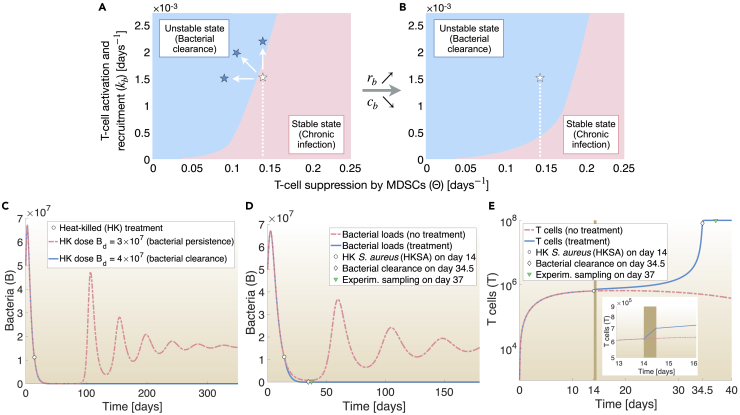
Model-driven insights for bacterial clearance (A and B) Based on the eigenvalues of the ODE system, the phase diagram was divided into stable states (pink) and unstable states (blue), which represent the physiological chronic infection and bacterial clearance, respectively. The white star represents the average position of an infected host during a chronic staphylococcal infection and was determined by the fitted values of parameters *k*_*b*_ and Θ (Table S1). Given the position of the infected host (white star), bacterial clearance is achieved (A) by an increase of T-cell activation and recruitment (*k*_*b*_) and/or decrease of T-cell suppression by MDSCs (Θ) (shown as blue stars) or (B) by increasing the proliferation rate of bacteria (*r*_*b*_) and/or reducing the bacterial containment by T cells (*c*_*b*_). (C-E) *In silico* treatment with HKSA. Initial infection was induced by setting the bacterial population equal to 5×10^7^ cells at day 0 in Equation 1. T-cell were set to 10^3^ at day 0 in Equation 2. HKSA-treatment was administered *in silico* at day 14 of infection (shown as white bullet) by adding the term *k*_*b*_*B*_*d*_ to the T-cell ODE at time t = perturbation day, for a 12-h perturbation window as explained in Equation 3 (STAR Methods). (C) A minimum dose of HKSA for resolving chronic *S. aureus* infection was estimated to be *B*_*d*_ = 4×10^7^ HKSA. *In silico* administration of 10^8^ HKSA, namely staphylococcal antigens, at day 14 of infection provides (E) quick boost of T cells (increase of parameter *k*_*b*_), as shown in brown, and (D) bacterial numbers are diminished to zero. Chronic *S. aureus* infection is resolved.

To explore perturbation strategies (treatments) that would destabilize the equilibrium between bacteria, T cells, and MDSCs, we varied values of *k*_*b*_ and Θ. These parameters, representing T-cell activation and recruitment by bacterial presence, and T-cell suppression by MDSCs, respectively, were specifically chosen because they play a key role in the establishment of chronic infection.^9^^,^^10^ Different values of *k*_*b*_ and Θ gave different eigenvalues for the ODE system (Equations 1 and 2), which were used to characterize the steady states (equilibria) of the mathematical model as unstable or stable (analytical forms are found in STAR Methods). Combining *in silico* all steady states in one phase diagram led to the distinguished areas of bacterial clearance and chronic infection (Figure 2A).

To gain more understanding of the dynamical system and suggest strategies for bacterial clearance, we next estimated the average position of an infected host on the phase diagram of bacterial clearance and chronic infection (Figure 2A, *white star*), using the values of parameters *k*_*b*_ and Θ as fitted previously (Table S1). We found that it lay in the region of chronic infection, yet close to the basin of attraction (region) of bacterial clearance. According to the phase diagram, we concluded that the resolution of chronic infection is achieved via either (a) relocation of the infected host from the chronic infection area toward the area of bacterial clearance (Figure 2A) by (i) increasing T-cell activation and recruitment (*k*_*b*_) and/or by (ii) decreasing T-cell suppression by MDSCs (Θ) or (b) via expansion of the bacterial clearance zone itself (Figure 2B) by (iii) increasing the proliferation rate of bacteria (*r*_*b*_) and/or (iv) by reducing bacterial containment by T cells (*c*_*b*_). These *in silico* results are in accordance with previous experimental studies reporting that brief suppression of immunity (namely *c*_*b*_ reduction) with cyclophosphamide^3^ or targeting MDSCs (namely Θ decrease)^34^ during *S. aureus* infection reduce bacterial burden. Altogether the model indicates that all four aforementioned perturbation categories (each of which can be implemented *in vivo* in various ways) can destabilize the dynamics of chronic infection so that *S. aureus* is cleared (Figure S1).

### Design of quantitative, model-driven experiments

Our *in silico* analysis suggested treatment strategies that were MDSC-targeting (Θ decrease), pathogen-targeting (*r*_*b*_ increase or *c*_*b*_ decrease), or host-directed (*k*_*b*_ increase). Experimental testing was essential to validate the model predictions. Since the validation of bacterial eradication would have immense importance for human therapeutics, we sought to test experimentally a treatment that would be easy to apply, while minimizing therapy-induced side effects.

MDSC-targeting drugs, such as gemcitabine or 5′Fluorouracil are chemotherapeutic agents, which are known to be associated with cytotoxicity, debilitate the recipients and they can bear severe side effects.^35^^,^^36^ They can simultaneously have a negative impact on healthy tissues and mature immune cells,^37^^,^^38^ which are indispensable components of immunity. Similarly, immunosuppression-inducing drugs (*c*_*b*_ decrease), such as cyclophosphamide, are also chemotherapeutic agents, hence they can be associated with cytotoxicity and concomitant serious side effects.^35^^,^^36^ Furthermore, chemotherapeutic drugs have been associated with damage in several organs. For example, gemcitabine can be associated with nephrotoxicity,^35^ while cyclophosphamide can be highly hepatotoxic.^35^ In an effort to save the kidney(s) from *S. aureus* as it is the aim of this study, it wouldn’t be reasonable to use drugs that have a serious potential to cause chemotherapy-induced side effects in the kidneys or other organs. It is worth pointing out, however, that a short course of cyclophosphamide in *S. aureus-*infected mice resulted in the bacterial reduction,^3^ hence corroborating experimentally our *in silico* results.

Despite ample research focusing on the development of antimicrobial drugs, there is surprisingly a large gap in the development of therapeutics that promote host defense during infection.^39^ Therefore, for our experimental testing, we wanted to investigate the immunostimulatory category of *k*_*b*_ increase (namely T-cell activation and recruitment). One of the most conventional and safe ways to boost *k*_*b*_ *in vivo*, which is also an established, widely used method for vaccine development, is via the administration of inactivated bacteria, namely antigens. In contrast to the potentially serious, therapy-induced side effects of chemotherapy, inactivated cells have been previously used by others to treat chronic *S. aureus* infection (furunculosis) with very mild side effects such as pain at the site of injection^7^ but also as a treatment for tuberculosis, for which scientists reported an excellent safety record that led to Phase III clinical trials in multiple countries around the globe.^40^^,^^41^ Such treatment could be extremely practical in human therapeutics, since pathogens causing renal abscesses in humans are typically isolated and cultured from each patient’s urine and/or blood samples for diagnostic purposes^21^^,^^42^^,^^43^^,^^44^ and could subsequently be easily inactivated (sterilized) to be used for treatment. We chose the inactivation of bacteria with heat over toxic chemicals, such as formalin. While inactivated bacteria in vaccines serve as prophylaxis from infection, their use as the treatment for ongoing chronic *S. aureus* infections in internal organs, such as the kidneys, is limited. Here we explored whether HK treatment during infection leads to bacterial clearance, as our *in silico* results suggested.

Since our aim was to resolve chronic *S. aureus* infection, the experimental perturbation (HK treatment) had to be carried out when the infection enters its chronic phase. As we have previously shown, inoculation with 3-7×10^7^ colony-forming units (CFU) of *S. aureus* results in chronic infection and renal abscesses, and by day 14 of infection T cells are already strongly suppressed by MDSCs.^9^^,^^10^ Therefore, the perturbation with HKSA was scheduled at day 14 after the initial infection with 5 × 10^7^ CFU of *S. aureus*. The physiological *k*_*b*_ increase via HK treatment was incorporated into the model with the addition of the term *k*_*b*_*B*_*d*_ to the T cells ODE on the day of treatment for a duration of 12 h (STAR Methods), where *B*_*d* =_ 10^8^ the dose of HKSA and *k*_*b*_ the activation and recruitment of T cells via HKSA assumed the same as for live bacteria during initial inoculation (Table S1, details in STAR Methods).

Numerical simulations for initial inoculation with 5 × 10^7^ *S. aureus* cells and HK treatment at day 14 of infection suggested that the minimum HKSA-dose required for bacterial clearance would be 4×10^7^ HKSA (Figure 2C). For our experiments, we opted for the amount of 10^8^ HKSA. To identify, on which day the infected mice would recover from infection to perform the experimental sampling, we followed the *in silico* results, which predicted bacterial clearance at day 34.5 of infection (Figure 2D). Because biological systems involve extrinsic and intrinsic stochasticity and therefore not all infected mice are synchronized in the same infection phase, the experimental sampling was set at day 37 of infection (Figure 2D). As a result of secondary exposure to *S. aureus*, HKSA-treatment initiates a cascade of inflammatory events, providing a concomitant, rapid boost in T-cell population (Figure 2E, *brown*), which is followed by further, a gradual increase of activated T cells as illustrated in our simulations (Figure 2E). Consequently, HKSA-treatment destabilizes to a sufficient extent the system dynamics of chronic infection and leads to bacterial clearance (Figures 2D and 2E).

### *In vivo* bacterial clearance after model-driven perturbation treatment with heat-killed *S. aureus*

**Figure 3:**
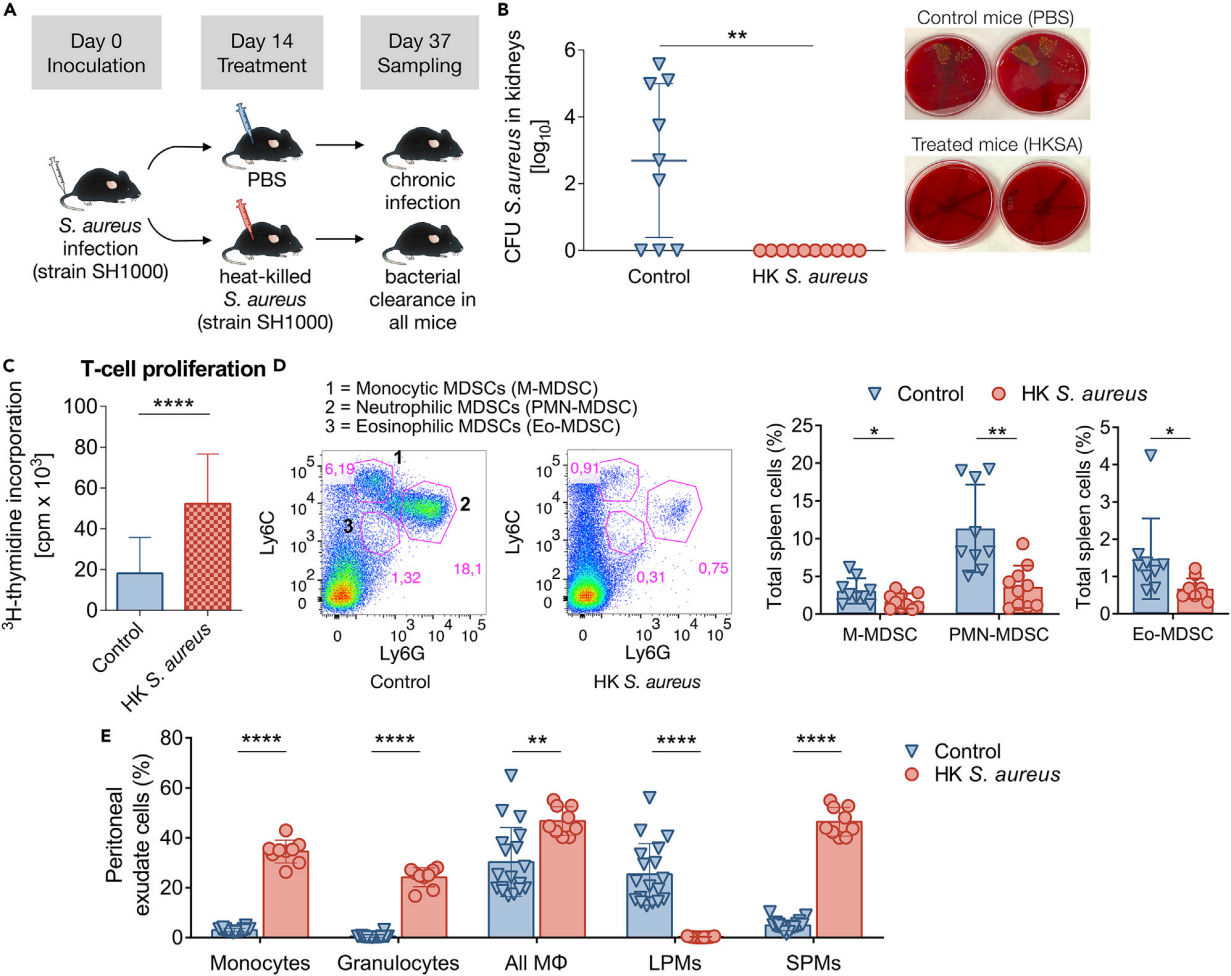
Bacterial burden and immune cells after treatment with heat-killed *S. aureus* (A) Experimental schema. C57BL/6 mice were intravenously inoculated with 5 × 10^7^ CFU of *S. aureus* and intraperitoneally treated with 10^8^ HKSA at day 14 of infection. Untreated (control) mice received PBS. Sampling was conducted at day 37 of infection. (B) Treatment with HKSA at day 14 of infection clears *S. aureus* in all chronically *S. aureus*-infected mice. Bacterial loads were determined in kidneys of untreated (control) and HKSA-treated mice at day 37 of infection. Kidneys were homogenized and plated on blood agar for colony formation and enumeration. n = 4-5 mice per group, two independent experiments. Data represent mean ± SD ∗∗p = 0.0018, Student’s *t* test. Right: Representative images of 10-fold serial dilutions on blood agar plates for the enumeration of viable *S. aureus* (golden-yellow colonies) in kidney homogenates from control (untreated) or HKSA-treated mice, showing high bacterial burden (bacterial persistence) or absence of bacteria (bacterial eradication), respectively. (C) Proliferative response of spleen T cells at day 37 of infection from chronically *S. aureus*-infected mice which received PBS or HKSA to *in vitro* stimulation with anti-CD3/anti-CD28. T-cell proliferation was measured by ^3^H-thymidine incorporation. n = 4-5 mice per group, two independent experiments. Data represent mean counts per minute (cpm) ± SD ∗∗∗∗p < 0.0001, Student’s *t* test. (D) HKSA-treatment reduces MDSCs. (left) Representative fluorescence-activated cell sorting (FACS) illustrating monocytic CD11b^+^Ly6C^+^Ly6G^low^, neutrophilic CD11b^+^Ly6C^low^Ly6G^+^ and eosinophilic CD11b^+^Ly6C^low^Ly6G^low^ MDSCs, and (right) percentage of each MDSC subset within the total spleen cell population of untreated (control) or HKSA-treated mice at day 37 of infection. n = 4-5 mice per group, two independent experiments. Data represent mean ± SD ∗p < 0.05 and ∗∗p < 0.01, Student’s *t* test. (E) Intraperitoneal HKSA-treatment at day 14 of infection induces acute inflammation. Peritoneal exudate cells were collected from chronically *S. aureus*-infected mice 12 h after the administration of PBS (control) or HKSA. Percentage of CD11b^+^Ly6C^+^ monocytes, CD11b^+^Ly6G^+^ granulocytes, CD11b^hi^F4/80^hi^ LPMs, CD11b^+^F4/80^low^ SPMs along with total MΦ population (LPMs plus SPMs) within the total peritoneal cell population is shown. n = 5-10 mice per group, two independent experiments. Data represent mean ± SD ∗∗p < 0.01, ∗∗∗∗p < 0.0001, Student’s *t* test.

Our next step was to provide proof-of-concept by validating our *in silico* predictions of bacterial clearance *in vivo*. For this purpose, C57BL/6 mice were infected intravenously with *S. aureus* strain SH1000. At day 14 of infection mice were treated intraperitoneally with HKSA, strain SH1000. Control mice received phosphate-buffered saline (PBS) (Figure 3A).

Our previous studies in chronically *S. aureus* SH1000-infected C57BL/6 mice have shown, using not only CFU counting but also by the visualization of luminescent bacteria, that *S. aureus* is progressively depleted from multiple sites and persists only in the kidneys.^10^^,^^45^ Therefore, at day 37 of infection, bacterial load quantification was performed in mice’s kidneys. The mathematical model’s predictions *in silico* (Figure 2D) were validated by our experiments *in vivo*: no *S. aureus* was found in the kidneys of any of the HKSA-treated mice (success percentage of 100%) (Figure 3B). In contrast, the majority of untreated animals remained infected with high bacterial burden (Figure 3B).

Progression of *S. aureus* infection from acute to chronic drives spleen T cells into T-cell dysfunction, which we previously found irreversible.^10^ T-cell dysfunction during chronic *S. aureus* infection is caused by the expansion and suppressive effect of all three MDSC subsets in spleen.^9^^,^^16^ Therefore, at day 37 of infection, we next investigated the effect of the HKSA-treatment on the proliferative response of T cells and on the MDSC populations. We found that HKSA-treatment restored the function of spleen T cells. Stimulation with anti-CD3/anti-CD28 antibodies showed that T cells were hyperresponsive and actively proliferated (Figure 3C). In contrast, spleen T cells of infected untreated animals remained hyporesponsive to TCR re-stimulation (Figure 3C). We further observed a significant reduction of all MDSC subsets in spleens of HKSA-treated mice, whereas MDSC populations remained high in spleens of infected, untreated (control) animals, which had received PBS (Figure 3D). Together, these findings reveal that HKSA-treatment during chronic *S. aureus* infection not only boosts T-cell function (namely *in vivo k*_*b*_ increase) but also has a previously unidentified potential to indirectly target the MDSCs (namely *in vivo* Θ decrease). Because MDSC-expansion and MDSC-mediated suppression on T cells are associated with the chronic but not acute phase of the infection^9^^,^^10^^,^^12^), these results suggest that acute inflammation caused by the administration of HKSA (see below) disrupts the balance of chronic infection, which sustains MDSCs, leading to natural MDSC depletion.

### Heat-killed *S. aureus* treatment during chronic *S. aureus* infection induces strong acute inflammation

Our *in silico* analysis suggested that destabilizing the system of chronic *S. aureus* infection by administering a sufficient amount of HKSA would lead to bacterial clearance. It was natural to expect that the insertion of staphylococcal antigens into the hosts via HKSA injection would trigger acute inflammation. To verify *in vivo* that the HKSA injection initiated acute inflammation during chronic *S. aureus* infection, we sampled peritoneal exudates 12 h after intraperitoneal HKSA-treatment and assessed differences in populations of macrophages, as well as infiltrating monocytes and granulocytes compared to untreated mice. It has been previously shown that two distinct subsets of macrophages exist in mouse peritoneal cavity (PerC), the CD11b^hi^F4/80^hi^ large peritoneal macrophages (LPMs) and the CD11b^+^F4/80^low^ small peritoneal macrophages (SPMs), and together are responsible for most of the phagocytosis happening in PerC.^46^^,^^47^ Under normal physiological conditions, LPMs are the predominant macrophage subset in PerC.^46^ However, under inflammatory conditions, the PerC environment changes drastically: LPMs disappear and SPMs become the major subset along with the substantial recruitment of SPM-precursors, the CD11b^+^Ly6C^+^ monocytes^46^ and of CD11b^+^Ly6G^+^ granulocytes (neutrophils). In accordance, we observed a significant increase in amounts of CD11b^+^Ly6C^+^ monocytes (∼10-fold higher) and CD11b^+^Ly6G^+^ granulocytes (neutrophils) (∼11-fold higher) in PerC of HKSA-treated mice, compared to untreated (control) mice (Figure 3E). LPMs were the predominant macrophage subset in chronically *S. aureus*-infected, untreated mice, indicating homeostatic conditions in PerC, whereas the treatment of chronically *S. aureus*-infected mice with HKSA resulted in the disappearance of LPMs and predominance of SPMs, indicating acute inflammation (Figure 3E). The total number of MΦ (LPMs plus SPMs) was increased in PerC of HKSA-treated mice (Figure 3E). These results suggest that treatment with HKSA during chronic *S. aureus* infection induced strong acute inflammatory responses.

### Non-antigen-specific heat-killed treatment

**Figure 4:**
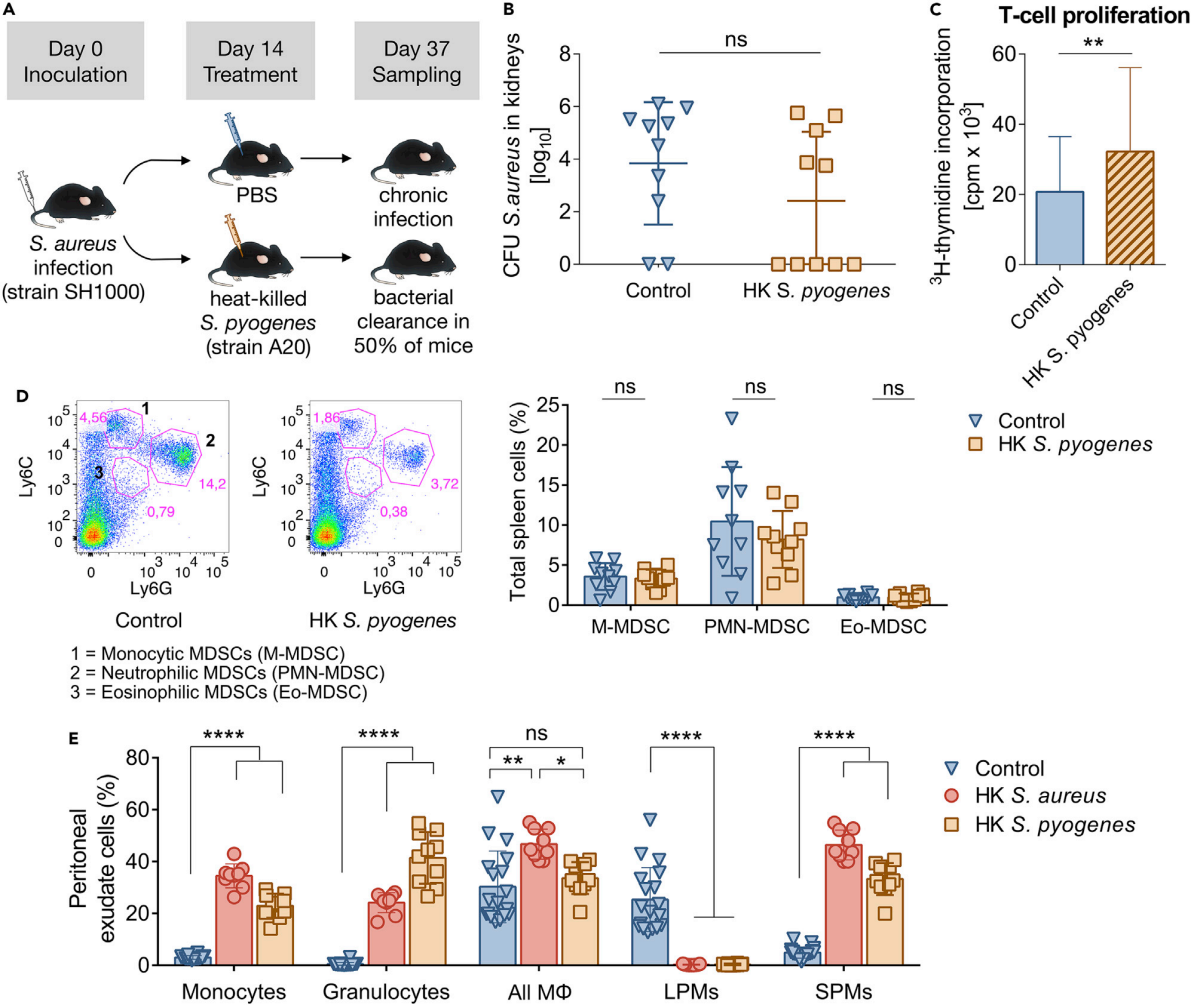
Bacterial burden and immune cells after treatment with heat-killed *S. pyogenes* (A) Experimental schema. C57BL/6 mice were intravenously inoculated with 5 × 10^7^ CFU of *S. aureus* and intraperitoneally treated with 10^8^ HKSP at day 14 of infection. Untreated (control) mice received PBS. Sampling was conducted at day 37 of infection. (B) Treatment with HKSP at day 14 of infection clears *S. aureus* in 50% of chronically *S. aureus*-infected mice. Bacterial loads were determined in kidneys of untreated (control) and HKSP-treated mice at day 37 of infection. Kidneys were homogenized and plated on blood agar for colony formation and enumeration. n = 5 mice per group, two independent experiments. Data represent mean ± SD, Student’s *t* test. (C) Proliferative response of spleen T cells at day 37 of infection from chronically *S. aureus*-infected mice which received PBS or HKSP to *in vitro* stimulation with anti-CD3/anti-CD28. T-cell proliferation was measured by ^3^H-thymidine incorporation. n = 5 mice per group, two independent experiments. Data represent mean counts per minute (cpm) ± SD ∗∗p = 0.0024, Student’s *t* test. (D) HKSP-treatment slightly reduces MDSCs in spleen. Representative fluorescence-activated cell sorting (FACS) illustrating monocytic CD11b^+^Ly6C^+^Ly6G^low^, neutrophilic CD11b^+^Ly6C^low^Ly6G^+^ and eosinophilic CD11b^+^Ly6C^low^Ly6G^low^ MDSCs (left), and percentage of each MDSC subset within the total spleen cell population of untreated (control) or HKSP-treated mice (right) at day 37 of infection. n = 5 mice per group, two independent experiments. Data represent mean ± SD, Student’s *t* test. (E) Intraperitoneal injection with HKSP at day 14 of infection induces acute inflammation. Peritoneal exudate cells were collected from chronically *S. aureus*-infected mice 12 h after the administration of PBS (control) or HKSP. Percentage of CD11b^+^Ly6C^+^ monocytes, CD11b^+^Ly6G^+^ granulocytes, CD11b^hi^F4/80^hi^ LPMs, CD11b^+^F4/80^low^ SPMs along with total MΦ population (LPMs plus SPMs) within the total peritoneal cell population is shown. n = 5-10 mice per group, two independent experiments. Data represent mean ± SD ∗p < 0.05, ∗∗p < 0.01, ∗∗∗∗p < 0.0001, one-way ANOVA with Tukey’s multiple comparisons test. Peritoneal exudate cells collected 12 h after HKSA-treatment at day 14 of infection are included for comparison.

The mathematical model suggested that treatment with HK *S. aureus* would resolve chronic *S. aureus* infection. Because MDSCs during chronic *S. aureus* suppress T cells, including antigen-specific T cells, we proceeded to assess experimentally whether the HK treatment (perturbation strategy) works in an antigen-specific manner. For reliable comparison with the experiments using HKSA, we maintained the experimental design of the aforementioned quantified HKSA protocols (namely same CFU of *S. aureus* for inoculation, HK-dose, routes of administration, times of treatment, and sampling) and only replaced HKSA with HK cells of a different bacterium. Therefore mice were inoculated with 5 × 10^7^ *S aureus*, however, they were treated intraperitoneally with 10^8^ HK *S. pyogenes* at day 14 of infection. Sampling was performed at day 37 of infection (Figure 4A). HKSP-treatment successfully cleared *S. aureus* in kidneys of 50% of chronically *S. aureus*-infected mice (Figure 4B), whereas the majority of control mice remained infected. Similarly to experiments with HKSA-treatment, we explored how HKSP-treatment affected T-cell proliferation and MDSC populations. Spleen T cells of HKSP-treated mice responded to stimulation with anti-CD3 plus anti-CD28 antibodies and proliferated significantly more than spleen T cells of untreated mice, which remained hyporesponsive (Figure 4C). In contrast to HKSA-treatment that led to considerable MDSC depletion, the reduction of MDSCs in HKSP-treated mice was insignificant compared to MDSCs in untreated mice (Figure 4D). It is worth noting, that in an individual level, each successfully HKSP-treated mouse (in which no *S. aureus* at all was found in its kidneys), had high T-cell proliferation along with a decrease in MDSC levels. However, in each of the unsuccessfully HKSP-treated mice milder T-cell proliferation was observed and the decrease of their MDSC levels was moderate.

As in the case of HKSA-treatment, peritoneal exudates were sampled 12 h after HKSP-treatment and confirmed acute inflammation. CD11b^+^Ly6C^+^ monocytes and CD11b^+^Ly6G^+^ granulocytes (neutrophils) increased significantly in PerC of HKSP-treated mice by ∼6-fold and ∼20-fold, respectively, compared to untreated mice (Figure 4E). LPM disappearance further confirmed acute inflammation induced by HKSP-treatment along with a significant increase of SPMs (Figure 4E). It is important to note that after HKSA-treatment, which cleared *S. aureus* in kidneys of all infected hosts, CD11b^+^Ly6C^+^ monocytes increased significantly, whereas after HKSP-treatment, which cleared *S. aureus* in kidneys of half of the infected hosts, CD11b^+^Ly6G^+^ granulocytes (neutrophils) increased significantly (Figure 4E).

These results confirmed that HK treatment with either *S. aureus* or *S. pyogenes* induces acute inflammation during chronic *S. aureus* infection. However, it was expected that HKSA-treatment would initiate stronger acute inflammation than HKSP-treatment, because HKSA was a re-exposure to *S. aureus*, while HKSP-treatment was a first-time exposure to *S. pyogenes* antigens. This is reflected by a significant increase of total MΦ (LPMs plus SPMs) in PerC after HKSA but not after HKSP-treatment compared to control, untreated mice (Figure 4E). Weaker acute inflammation after HKSP- than HKSA-treatment was also indicated by less reduction of MDSCs in spleens (Figures 3D and 4D), which are sustained in conditions of chronic (but not acute) inflammation^12^ in addition to almost 2-fold lower proliferative response of spleen T cells in HKSA-treated compared to HKSP-treated mice (Figures 3C and 4C). These results combined can explain bacterial clearance in 50% of HKSP-treated animals in comparison to bacterial clearance in 100% of HKSA-treated animals and are in accordance with previous studies, suggesting that monocytes and macrophages promote *S. aureus* clearance^34^ and that after antigen immunization in PerC, SPMs migrate to lymph nodes where they activate T cells.^47^^,^^48^

Because treatment with 10^8^ HKSP induced weaker acute inflammation and T-cell stimulation than treatment with 10^8^ HKSA, we hypothesized (i) that treatment with higher HKSP-dose (more antigens) would naturally induce stronger acute inflammation (including more CD11b^+^Ly6C^+^ monocytes and SPMs), hence more indirect depletion of MDSCs and more activated T cells, leading to bacterial clearance in all HKSP-treated mice. It is also commonly known that secondary exposure to antigens elicits a faster immune response, meaning that HKSA-treatment which was a re-exposure to staphylococcal antigens, triggered a faster immune response compared to HKSP-treatment, which was a first-time exposure to streptococcal antigens. Therefore, for HKSP-dose the same as HKSA-dose, HKSP-treatment would require longer time to induce immune response relatively as strong as that induced by HKSA. Consequently, although bacterial clearance in 50% of HKSP-treated mice at day 37 of infection, when experimental sampling was conducted, was not statistically significant (Figure 4B), we hypothesized (ii) that at a later time-point HKSP-treatment could eventually clear *S. aureus* in all hosts. To investigate our hypotheses (i) and (ii), we reduced *in silico* the value of parameter *k*_*b*_ in the treatment term *k*_*b*_*B*_*d*_ by two or three times to reflect the weaker T-cell stimulation by HKSP compared to HKSA and we estimated the day of clearance (i) for varying HKSP-doses *B*_*d*_ administered at day 14 of infection and (ii) for 10^8^ HKSP (*B*_*d*_) administered at day 14 of infection. Our simulations suggested that *S. aureus* may be cleared in kidneys of all HKSP-treated mice (namely 100% success) (i) if higher HKSP-dose (>10^8^) is administered and sampling is conducted on the day of sampling, namely day 37 of infection (Figure S2), or (ii) if HKSP-dose = 10^8^ is administered and sampling is conducted approximately 10 days or later than day 37 of infection (Figure S2).

### *In silico* implications to potential clinical implementation

Lastly, we conducted an *in silico* investigation (STAR Methods) of a published clinical trial for the treatment of chronic *S. aureus* furunculosis using inactivated *S. aureus* that was administered in multiple doses.^7^ The conventional administration of injections with inactivated bacteria is based on the belief that repeated vaccination could work more efficiently. Here, we employed *in silico* the analogous protocol that has been administered in humans (STAR Methods, suspensions IV-VI, 19 injections). Our simulation results indicated that repeated injections with increasing doses of killed *S. aureus* may not be able to render the eradication of *S. aureus* (Figure S3A). We also explored the case when not only more injections are given (25 in total) but also with high fixed dose (almost 5-fold higher than the highest dose of suspensions IV-VI) and found that such treatments can remain unsuccessful in eliminating the infection (Figure S3A). Our *in silico* results suggested that the doses of inactivated *S. aureus* that were used in the clinical trial may have been too low for complete *S. aureus* clearance and that a single injection with high dose could have been successful, thus making multiple low-dose injections redundant (Figures S3 and S4). Furthermore, our results showed that in case time intervals between injections need to increase, (e.g. schedules of doctors), then higher HK doses are required for bacterial clearance (Figure S4B).

Even though treatments with insufficient-for-bacterial-clearance doses of inactivated *S. aureus*, such as the doses given to patients with furunculosis, cannot confer complete clearance of bacteria, patients with furunculosis still reported improvement. In accordance, *in silico* administration of low HK doses showed that although they do not lead to complete bacterial clearance in the hosts, they alleviate chronic infection by providing remission (Figure S4D). In fact, the administration of HK treatment as early as possible leads to longer remission of the infection (Figure S4D).

We further investigated how the administration time of a HK-injection affects the clinical outcome. For middle-sized doses the day of administration is crucial for the outcome of chronic infection, since it can provide complete bacterial clearance if given as early as possible (namely after an early and accurate diagnosis, which is often challenging for renal abscesses) or only provide temporary remission without clearing the infection (Figure S4E). These results highlight the urgency of treating chronic *S. aureus* infections and renal abscesses as early as possible, which is, due to diverse and non-specific symptomatology, often compromised by improper and/or time-consuming treatments.^21^^,^^42^

## Discussion

At present, no treatment has proved to be completely effective in resolving chronic *S. aureus* infections. There is no vaccine against *S. aureus* infections because all clinical trials have failed.^49^ The development of new antibiotics would only be a temporary solution, until the bacterium develops antibiotic resistance. Renal abscesses by *S. aureus* are considered rare but with potentially severe complications. Recent publications identify antibiotic-resistant *S. aureus* in organs that have not been conventionally infected before, such as kidneys. The studies report renal abscesses by Panton-Valentine leukocidin-producing *S. aureus*,^43^ which can cause leukocyte destruction and tissue necrosis, including Panton-Valentine leukocidin-positive *S. aureus* abscess in a transplanted kidney,^50^ renal abscess in healthy children,^51^ renal abscess in previously healthy nursing staff after an outbreak of MRSA infections in a tertiary care unit,^44^ as well as bilateral renal abscesses that required bilateral nephrostomy^52^ or bilateral nephrectomy as an extreme intervention to prevent death.^21^ Such reports highlight that treating infections in organs, where the medical community is not traditionally used to encountering, is very challenging and often misdiagnosed, therefore requiring prompt intervention once identified.^42^ Furthermore, the current medical arsenal of antibiotics, MDSC-targeting drugs or surgery, is insufficient, ineffective or inadvisable due to bacteria-resistance mechanisms, cytotoxicity of drugs, inability to drain abscesses because of their location in internal organs or the high-risk medical status of patients. Consequently, new, less complicated ways of treatment against *S. aureus* infections are absolutely essential to find. We here reported bacterial clearance in all chronically *S. aureus*-infected mice with renal abscesses, using heat-killed *S. aureus* treatment, as quantified by our mathematical model. Although inactivated *S. aureus* has been used to treat soft skin infections^7^ this is, to our knowledge, the first time heat-killed *S. aureus* is used to treat renal abscesses.

Conventional vaccination with inactivated microorganisms always requires multiple doses to ensure prophylaxis. Hence, it is believed that inactivated microorganisms, when used as treatment, should also be administered in multiple doses to be efficient.^7^ Here we provided evidence that treatment with one HK-injection alone can confer bacterial clearance (Figures 2D and 3B). This is an important finding, because it means that a single injection with the right HK-dose, besides being extremely practical to implement in human therapeutics, could suffice to break the chronic phase of infection and lead to bacterial clearance, without the need for antibiotics that multidrug-resistant *S. aureus* can evade, or surgery for abscess drainage, or cytotoxic MDSC-targeting drugs. A practical aspect of this treatment is that urine and/or blood samples are routinely obtained from each patient to determine via cultures the infection-causative pathogen,^21^^,^^42^^,^^43^^,^^44^ which could subsequently be heat-killed (sterilized) and used for treatment. Regarding safety, the administration of inactivated *S. aureus* (initially isolated from suppurative skin lesions of individual patients) as the treatment of staphylococcal skin infection in human patients had only minor side effects, such as temporary pain at the site of injection.^7^ In accordance, our treatments with HKSA or HKSP were well-tolerated. Treated animals did not exhibit any signs of side effects or discomfort. They were active, alert, observant of their environment, with eyes wide open, fur laying flat, and maintained normal body weight and appetite. Consequently, if further research in human patients with renal abscesses corroborates the efficacy and safety, HK treatments bear the potential to become easily produced, easily stored, cost-effective, and safe treatments in medicine.

The recently identified Eo-MDSC inhibit, as other MDSCs, T-cell proliferation during chronic *S. aureus* infection.^16^ Our results agree with previous results on the suppressive effect of Eo-MDSC on T cells, since high Eo-MDSC numbers in control (untreated) mice contributed to high T-cell suppression that was reduced as a result of reduced Eo-MDSC after HKSA-treatment (Figures 3C and 3D). More Eo-MDSC have been directly linked with more CFU of *S. aureus*.^16^ In accordance, our results showed that a significant decrease of Eo-MDSC contributed to bacterial clearance (Figures 3B and 3D). Additionally, we here revealed a previously unidentified approach for the depletion of Eo-MDSC that was achieved in an indirect way, in contrast to cytotoxic agents, which directly target MDSC populations and bear side effects.

Our mathematical model aimed to predict variables that could transition a chronic *S. aureus* infection to an active inflammatory state to promote bacterial clearance. Our experiments *in vivo* verified that perturbation with HKSA initiates acute inflammation during the chronic establishment of *S. aureus* infection, showing a significant increase in amounts of CD11b^+^Ly6C^+^ monocytes, CD11b^+^Ly6G^+^ granulocytes (neutrophils) and CD11b^+^F4/80^low^ SPMs in HKSA-treated mice (Figure 3E). Our previous study has shown that T-cells from chronically *S. aureus*-infected mice remained hyporesponsive after *in vitro* stimulation with either heat-killed *S. aureus* or anti-CD3 plus anti-CD28.^10^ This indicates that it is not the heat-killed treatment per se that resolved the infection, but rather the mathematically driven concept of sufficiently destabilizing the system dynamics *in vivo*. One of the strategies for the destabilization of the chronic infection system, as shown in this study, was achieved via the administration of HKSA that caused acute inflammation and subsequently created a new cascade of inflammatory events (including T-cell boost), resulting in bacterial clearance.

For the experiments regarding antigen-specificity of the treatment, *S. pyogenes* was chosen because together with *S. aureus* they are the two most common gram-positive cocci of medical significance.^53^ Delivery of 10^8^ HKSP cleared *S. aureus* in 50% of animals, suggesting that a portion of the effects of the HK treatment are antigen-independent and that a non-antigen specific HK treatment may be completely effective if combined with other perturbation strategies (antigen-specific or not). Even a higher HKSP-dose alone could have potentially resolved the chronic infection in all HKSP-treated animals (Figure S2). Interestingly, we found that other studies have used a similar approach to treat chronic *Mycobacterium tuberculosis* infections with heat-killed *Mycobacterium vaccae,* especially in patients, in whom previous treatment with antibiotics and/or chemotherapy had failed. Consistent with our results, they reported a therapeutic effect that cures patients (success percentages varying), along with an increase of T cells and an exemplary safety record even in patients co-infected with HIV. The copious advantages of heat-killed *M. vaccae* treatments led to extensive Phase III clinical trials in patients in a plethora of countries around the globe.^40^^,^^41^ Taking all the above into account, our results with *S. pyogenes* could be a pioneer for chronic *S. aureus* infections because they suggest that chronic *S. aureus* infections may be successfully treated with other *S. aureus* strains or even other bacteria, for example *S. pyogenes*. If the presented results are corroborated by further evidence and hold in humans, a standardized cocktail of the most frequent organisms causing renal abscesses e.g. *S. aureus, Escherichia coli (E. coli)* could be added to hospital stocks for the immediate treatment of patients afflicted with renal abscesses. In addition, these results could also help current efforts toward vaccine development for protection against *S. aureus*, since antigens from other cocci/bacteria could be incorporated for vaccine testing.

Some diversity in the bacterial loads of control mice in all experiments *in vivo* was observed (Figures 3B and 4B). All control mice were infected with *S. aureus* but did not receive heat-killed treatment. It was observed that in some of them *S. aureus* was cleared. Such variability can be occasionally observed since (i) individual immune responses of mice can vary greatly and (ii) biological systems are inherently stochastic. However, chronic infection and associated renal abscesses caused by the same *S. aureus* strain as used here have been amply studied and the dynamics of chronicity *in vivo* were published already years ago.^9^^,^^10^ This observed behavior is explained mathematically in Figure 2A: The average position of an infected animal in the phase diagram (*white star*) lies in the basin of attraction of chronic infection (Figure 2A, *pink area*) but is very close to the basin of attraction of bacterial clearance (Figure 2A, *blue area*), indicating that a small portion of infected mice could spontaneously eliminate *S. aureus*. Mathematically, points that lie close to the boundary separating two basins of attraction could end up in the other attractor domain, namely the region of bacterial clearance, after stochastic perturbations (possibly happening during the acute phase of the infection). Most essential is, however, the effectiveness of the treatment because renal abscesses are often misdiagnosed, hence associated with high risk of mortality,^42^ and once correct diagnosis takes place, the patients might already experience organ damage and be in life-threatening conditions, whose lives could be saved because of this treatment.

It is worth pointing out that the four *in silico*-suggested, therapeutic categories could be achieved *in vivo* via a variety of treatments. For our experimental testing, we singled out the immunostimulatory category of T-cell activation and recruitment (*k*_*b*_ increase) and tested it *in vivo* with the use of heat-killed bacteria. However, T-cell activation and recruitment could be also achieved *in vivo* via other immune response-stimulating factors, such as bacterial surface factors, LPS, toxoids. Such therapeutic interventions could be investigated in subsequent studies for their advantages and disadvantages, such as efficacy, safety, and practicality, and be compared to heat-killed treatments.

In the present study, our mathematical model provides a guideline for the specific experimental set-up of one HKSA-dose (10^8^) at day 14 of infection and harvest at day 37 of infection (Figure 1, Figure 2, Figure 3, Figure 44, black square). In Figures S4A-S4C, the model further suggests a plethora of unique, therapeutic HKSA-treatment protocols with varying HK doses, number of HK-injections and time intervals between HK-injections (each blue bullet point). However, to further assess the reliability and accuracy of the model, more experimental evidence is needed. Future work could be to experimentally test additional, model-quantified treatment protocols by changing individual or several variables of the treatment setting (e.g. time of treatment, HK dose and so forth), or possibly even investigate experimentally the validity of model-driven treatment protocols that do not predict bacterial clearance. If they, too, are verified experimentally, the model’s suggested variety of quantified protocols could offer important flexibility for future treatments or clinical trials because they could comply with the scientific and social circumstances, for instance working hours of doctors, sensitivity of individuals to high HK doses, or restrictions in animal permits.

It is important to note that the mouse model of chronic *S. aureus* infection used in this study has been very well characterized in previous studies in terms of bacterial loads across organs using not only CFU counting but also by the visualization of luminescent bacteria using the Xenogen Vivo Vision IVIS 200 system.^10^^,^^45^ In these studies, the kidneys were found to be the main organ target for *S. aureus* persistence. These previously published studies combined with the detection of zero bacteria in any of the serial dilutions of kidney homogenates following HKSA or HKSP treatment in this study highly suggest that HKSA-treatment (in all treated mice) or HKSP treatment (in 50% of treated mice) can remove all bacteria from the system, thus lead to a potential cure.

The results of this study are specified to C57BL/6 female mice; however, new *in silico* therapeutic protocols can be obtained when fitting the parameters of the mouse line or human of interest. Most importantly, the simple structure of our model allows its easy adjustment for chronic infections caused by other bacteria or staphylococcal strains, which could suggest therapeutic protocols against other strain- or bacteria-specific infections, including chronic osteomyelitis, *S. aureus* biofilms on medical implants that are devastating the clinics, renal abscesses caused by other bacteria such as *E. coli*, or infections in other internal organs. Future studies could be such adjustment of model parameters for other bacteria or staphylococcal strains, such as the MRSA strain USA300 or the clinical isolate strain 6850, and subsequent experiments based on model-driven, strain-specific treatment protocols for verification.

We here report the eradication of *S. aureus* in chronically infected kidneys without any use of antibiotics, cytotoxic MDSC-targeting drugs, or surgical procedures for abscess drainage. Our study provides guidelines for a novel treatment protocol for chronic *S. aureus* infection by repurposing heat-killed treatments, directed by our mathematical model. Unlike conventional heat-killed administration, which is used as prophylaxis against infections in humans and sometimes animals, we used this method as a treatment during ongoing chronic infection. Treatments that are directed at promoting the immune response during an ongoing infection are lacking. Our work could pave the way and provide a new scientific perspective toward treatments that do not aim at targeting the infective agent (such as antibiotics), but rather boost the host’s own immune defense to resolve the infection.

### Limitations of the study

The MDSCs were not described in the mathematical model as a dynamical variable but rather the known T-cell suppression by MDSCs was incorporated in the model as parameter Theta. This is because: (i) there is a lack of biological knowledge regarding the exact interactions of different immune populations and all MDSC populations, apart from the fact of T-cell suppression. Therefore, we would have required additional assumptions with little biological back-up. (ii) MDSCs are heterogeneous and each of the so far three known populations evolves differently in time and can depend on the strain or the bacterium causing infection. Temporal kinetics of MDSCs during infection would have been required for exact parameter calibration. (iii) Increasing the complexity of the model could reduce the parameter identifiability and our fitting ability. Consequently, the mathematical model can predict the eradication of bacterial cells (which was the aim of our study) but cannot predict *in silico* any changes in MDSC numbers, which we observed in our *in vivo* experiments following the heat-killed treatment.

## STAR★Methods

### Key resources table

**Table:** 

REAGENT or RESOURCE	SOURCE	IDENTIFIER
**Antibodies**		

rat anti-mouse CD16/CD32	BD Biosciences	Cat #747949
rat anti-mouse CD11b-PE/Cy7	BioLegend	Cat. #101216
rat anti-mouse Ly6C-APC	BioLegend	Cat. #128016
rat anti-mouse Ly6G-PE	Miltenyi Biotec	Cat. #130-123-712
rat anti-mouse F4/80-PE	BD Biosciences	Cat #567893
Anti-mouse CD3	Sigma-Aldrich	Cat. #SAB4700048
Anti-mouse CD28	Sigma-Aldrich	Cat. #SAB4700739

**Bacterial and virus strains**		

*S. aureus* strain SH1000	Jonsson et al.^54^	rsbU^+^ derivative of strain 8325-4
*S. pyogenes* strain A20	German Culture Collection	DSM 2071

**Chemicals, peptides, and recombinant proteins**		

^3^H -Thymidine	Amersham, Buchler, Germany	N/A

**Deposited data**		

Raw experimental data	This paper (Mendeley)	https://doi.org/10.17632/36xvmr4mjj.1

**Experimental models: Organisms/strains**		

C57BL/6 female mice	Harlan-Winkelmann (Envigo, Netherlands)	N/A

**Software and algorithms**		

Matlab R2019a	Mathworks	www.mathworks.com
FlowJo Software	BD Biosciences	https://www.flowjo.com/solutions/flowjo/downloads/
GraphPad Prism 7.0 Software	GraphPad software Inc.	https://www.graphpad.com/scientific-software/prism/

### Resource availability

#### Lead contact

Request for additional information should be directed to Prof. Dr. Haralampos Hatzikirou (Email: haralampos.hatzikirou@ku.ac.ae).

#### Materials availability

All data that were generated are included in the manuscript. This study did not generate new unique reagents.

### Method details

#### Mathematical model

The mathematical model was implemented and simulated in MATLAB, see www.mathworks.com.

#### Fitting curves and standard deviation of parameters

The unknown parameters in the model were estimated in three steps. First, the carrying capacity of bacteria *κ* was estimated based on our previously reported experimental results from B and T cells-deficient RAG2^−/−^ mice (only innate immunity present), which were initially infected with 7 × 10^7^ CFU of *S. aureus* strain SH1000 (same strain as used in the study here) and showed a nearly constant level of *S. aureus* in kidneys from day 7 till day 56.^10^ The mean of these data points was taken as the bacterial carrying capacity. The carrying capacity of T cells (parameter $K_{T}$) was assumed to be of the same order of magnitude as the bacterial carrying capacity. Secondly, the growth rate of bacteria was estimated by solving the logistic growth equation $\frac{dP}{\mathrm{d}t}=rP\left(1-\frac{P}{\kappa }\right)$ for r

$$r=\;\frac{ln\left(\frac{P\left(\kappa -P_{0}\right)}{P_{0}\left(\kappa -P\right)}\right)}{t}$$

where *κ* is the bacterial carrying capacity, *P*_0_ is the initial inoculation number of bacteria and P is the bacterial CFU at time t. Our previous experimental data showed that the bacterial loads in B and T cells-deficient RAG2^−/−^ mice reached 75% of the carrying capacity at day 2 and fluctuated afterwards, after intravenous inoculation with 7 × 10^7^ CFU of *S. aureus*. Assuming that the bacterial load reached 75% of the carrying capacity by day 1 or day 2, we determined the high and low boundary of *r* to be 0.636 and 0.318 days^−1^, respectively. The average of the low and high boundary was taken as the bacterial growth rate *r*_*b*_ of the model. Finally, our previously reported data of bacterial loads and absolute numbers of T cells in time from immunocompetent mice with chronic *S. aureus* SH1000-infection and renal abscesses^10^ were used to fit the rest of unknown parameters. The fitting process used a Markov Chain Monte Carlo version of Differential Evolution algorithm.^55^ Parameter *c*_*T*_, representing T-cell suppression locally (namely at the site of infection which is the kidneys for the chronic infection here) was estimated to be equal to 2.2204 × 10^−16^during fitting, hence much smaller than other parameters, in particular parameter Θ (representing T-cell suppression by MDSCs systemically, namely distant from the site of infection, such as in spleen). We tested, in a second study, the possibility of *c*_*T*_ = 0, because *c*_*T*_ (order of magnitude of −16) is multiplied by *B(t)* and *T(t)* in the local immunosuppression term *c*_*T*_*B*(*t*)*T*(*t*), both of which have a maximum order of magnitude of 8 (values for carrying capacity of bacteria and T cells are shown in Table S1). The simulation results remained the same, independently of *c*_*T*_ ≠ 0 or *c*_*T*_ = 0 (Figure S5); therefore we concluded that *c*_*T*_ is zero and that the main T-cell suppression by MDSCs is exerted systemically. This deduction is in accordance with our previous experimental reports which showed major T-cell suppression by MDSCs in spleens of mice that had chronic *S. aureus* infection in kidneys.^9^^,^^16^ Fitted parameter values are shown in Table S1. It is important to note that these parameter values are specific for chronic infections by *S. aureus* strain SH1000 and can change (including the value of *c*_*T*_), when adjusting the model to predict therapeutic protocols for chronic infections caused by other *S. aureus* strains or other bacteria.

#### Scaling the model

For the analytical solutions and analytical stability analysis the calculations were made feasible by using an analytically amenable ODE model, which approximates the original ODE as described in Equations 1 and 2.

With the following change of variables we non-dimensionalize the ODE model (1)-(2):

$$B=\beta_{0}\xi ,\;T=c_{0}\psi ,\;t=t_{0}\tau .$$

$$\Rightarrow \frac{dB\left(t\right)}{dt}=\frac{\beta_{0}d\xi }{t_{0}d\tau }=r_{b}\beta_{0}\xi -\frac{r_{b}}{\kappa }\beta_{0}^{2}\xi^{2}-c_{b}c_{0}\psi \beta_{0}\xi$$

$$\Rightarrow \frac{d\xi }{\mathrm{d}\tau }=r_{b}t_{0}\xi -\frac{r_{b}}{\kappa }t_{0}\beta_{0}\xi^{2}-c_{b}t_{0}c_{0}\xi \psi .$$

We impose that the coefficients of $\xi ,\xi^{2}$ and ξψ are equal 1. Then

(Equation S1) $$t_{0}=\frac{1}{r_{b}},\;\beta_{0}=\kappa ,\;c_{0}=\frac{r_{b}}{c_{b}}\text{.}\;$$

Therefore the new, non-dimensionalized equation for bacteria is

(Equation S2) $$\frac{d\xi }{d\tau }=\xi -\xi^{2}-\xi \psi$$

For the equation $\overset{\cdot }{T}\left(t\right)$ we have the non-dimensionalized calculations:

$$\frac{dT\left(t\right)}{dt}=\frac{c_{0}d\psi }{t_{0}d\tau }=r_{t}c_{0}^{2}\psi^{2}+k_{b}\beta_{0}\xi -c_{T}\beta_{0}\xi c_{0}\psi -\text{Θ}c_{0}\psi$$

$$\Rightarrow \frac{d\psi }{d\tau }=r_{t}t_{0}c_{0}\psi^{2}-\text{Θ}t_{0}\psi -c_{T}\beta_{0}t_{0}\xi \psi +\frac{k_{b}\beta_{0}t_{0}}{c_{0}}\xi$$

Substitution of the $t_{0},\beta_{0}$ and $c_{0}$ (found in Equation (S1)) gives the scaled equation for T:

(Equation S3) $$\frac{d\psi }{d\tau }=\alpha \psi^{2}+\beta \xi -\gamma \xi \psi -\delta \psi$$

(Equation S4) $$\text{where}\;\alpha =\frac{r_{t}}{c_{b}},\beta =\frac{\kappa k_{b}c_{b}}{r_{b}^{2}},\gamma =\frac{\kappa c_{T}}{r_{b}},\delta =\frac{\text{Θ}}{r_{b}}\text{.}$$

#### Calculation of equilibrium points

From Equation S2 we have:

$$\xi -\xi^{2}-\xi \psi =0\Rightarrow \xi =0,\;or\;\xi =1-\psi$$

• For ξ=0 in Equation S3 we have:

$$\frac{d\psi }{d\tau }=0\Rightarrow \alpha \psi^{2}-\delta \psi =0\Rightarrow \psi_{1}=0,\text{or}\;\psi_{2}=\frac{\delta }{\alpha }$$

• For ξ=1−ψ in Equation S3 in steady state we conclude that:

(Equation S5) $$\psi_{3,4}=\frac{\beta +\gamma +\delta \pm \sqrt{{\left[-\left(\beta +\gamma +\delta \right)\right]}^{2}-4\beta \left(\alpha +\gamma \right)}}{2\left(\alpha +\gamma \right)}\;$$

Consequently the system has four equilibrium points in total:

• $\left(\xi_{1},\psi_{1}\right)=\left(0,0\right)$
• $\left(\xi_{2},\psi_{2}\right)=\left(0,\frac{\delta }{\alpha }\right)$
• $\left(\xi_{3,4},\psi_{3,4}\right)=\left(1-\psi_{3,4},\psi_{3,4}\right)$ where $\psi_{3,4}$ is as shown above.

#### Existence of $\psi_{3,4}$

Equilibrium points $\psi_{3,4}$ exist only when $\Delta ={\left(\beta +\gamma +\delta \right)}^{2}-4\beta \left(\alpha +\gamma \right)\geq 0$, i.e. only when $\psi_{3,4}$ have no imaginary part. Equivalently

$$\;{\left(\beta +\gamma +\delta \right)}^{2}-4\beta \left(\alpha +\gamma \right)\geq 0$$

$$\beta^{2}+\gamma^{2}+\delta^{2}+2\beta \gamma +2\beta \delta +2\gamma \delta -4\alpha \beta -4\beta \gamma \geq 0\;\left(\pm 4\beta \delta \right)$$

$$\beta^{2}+\gamma^{2}+\delta^{2}-2\beta \gamma -2\beta \delta +2\gamma \delta -4\beta \left(\alpha -\delta \right)\geq 0$$

(Equation S6) $${\left(-\beta +\gamma +\delta \right)}^{2}-4\beta \left(\alpha -\delta \right)\geq 0$$

According to the sign of the term (α−δ) in condition (S6), we investigate when the equilibrium points $\psi_{3,4}$ exist.

• If α−δ≤0⇒δ≥a then the equilibria $\psi_{3,4}$ exist.
• If 0<δ<α then we reformulate Δ as follows:

$$\Delta =\left(-\beta +\gamma +\delta -2\sqrt{\beta }\sqrt{\alpha -\delta }\right)\cdot \left(-\beta +\gamma +\delta +2\sqrt{\beta }\sqrt{\alpha -\delta }\right)$$

a) If −β+γ+δ≥0 then for existence of $\psi_{3,4}$ we require

$-\beta +\gamma +\delta -2\sqrt{\beta }\sqrt{\alpha -\delta }\geq 0$. Then:

$$-\beta +\gamma +\delta -2\sqrt{\beta }\sqrt{\alpha -\delta }\geq 0\Rightarrow \beta -\left(\gamma +\delta \right)+2\sqrt{\beta }\sqrt{\alpha -\delta }\leq 0$$

$${\sqrt{\beta }}^{2}+2\sqrt{\beta }\sqrt{\alpha -\delta }-\left(\gamma +\delta \right)\leq 0$$

$$\Rightarrow \sqrt{\beta }\leq \frac{-2\sqrt{\alpha -\delta }\pm \sqrt{4\left(\alpha -\delta \right)-4\left[-\left(\gamma +\delta \right)\right]}}{2}$$

$$\sqrt{\beta }\leq -\sqrt{\alpha -\delta }+\sqrt{\alpha +\gamma }$$

Note: The solution $\sqrt{\beta }\leq -\sqrt{\alpha -\delta }-\sqrt{\alpha +\gamma }$ is rejected because by definition $\sqrt{\beta }\geq 0$.

b) If −β+γ+δ≤0 then for existence of $\psi_{3,4}$ we require $-\beta +\gamma +\delta +2\sqrt{\beta }\sqrt{\alpha -\delta }\leq 0$. Then:

$$-\beta +\gamma +\delta +2\sqrt{\beta }\sqrt{\alpha -\delta }\leq 0\Rightarrow \beta -\left(\gamma +\delta \right)-2\sqrt{\beta }\sqrt{\alpha -\delta }\geq 0$$

$${\sqrt{\beta }}^{2}-2\sqrt{\beta }\sqrt{\alpha -\delta }-\left(\gamma +\delta \right)\geq 0$$

$$\Rightarrow \sqrt{\beta }\geq \frac{2\sqrt{\alpha -\delta }\pm \sqrt{4\left(\alpha -\delta \right)-4\left[-\left(\gamma +\delta \right)\right]}}{2}$$

$$\sqrt{\beta }\geq \sqrt{\alpha -\delta }+\sqrt{\alpha +\gamma }$$

Note: The solution $\sqrt{\beta }\geq \sqrt{\alpha -\delta }-\sqrt{\alpha +\gamma }$ is rejected for $\sqrt{\alpha -\delta }-\sqrt{\alpha +\gamma }>0$ because:

$$\sqrt{\alpha -\delta }-\sqrt{\alpha +\gamma }>0\;\Rightarrow \;\sqrt{\alpha -\delta }>\sqrt{\alpha +\gamma }\;$$

$${\left(\sqrt{\alpha -\delta }\right)}^{2}>{\left(\sqrt{\alpha +\gamma }\;\right)}^{2}\;\Rightarrow \;\alpha -\delta >\;\alpha +\gamma \;\Rightarrow \;-\delta >\gamma$$

while by definition δ>0 and *γ* *>* 0, hence −δ<0<γ⇒−δ<γ.

#### Local stability analysis of equilibrium points

Jacobian Matrix J = $\left[\begin{array}{l} \frac{\partial \dot{\xi }}{\partial \xi }\;\frac{\partial \dot{\xi }}{\partial \psi }\\ \frac{\partial \dot{\psi }}{\partial \xi }\;\frac{\partial \dot{\psi }}{\partial \psi } \end{array}\right]=\left[\begin{array}{ll} 1-2\xi -\psi & -\xi \\ \beta -\gamma \psi & 2\alpha \psi -\gamma \xi -\delta \end{array}\right]$

Evaluation of Jacobian matrix in the trivial equilibrium point $\left(\xi_{1},\psi_{1}\right)$:

(Equation S7) $$J\left(0,0\right)=\left[\begin{array}{ll} 1\; & 0\\ \beta & -\delta \end{array}\right]\Rightarrow |J\left(0,0\right)|=-\delta <0$$

From linear algebra, it is known that if J is a 2 × 2 matrix and λ_1_, λ_2_ its eigenvalues, then the determinant of J, |J| = λ_1_ × λ_2_. Since the determinant of the Jacobian matrix in Equation S7 is negative (i.e. det = -*δ* <0), it means that the eigenvalues are of a different sign, and hence the trivial equilibrium point is unstable (i.e. saddle point, which is always unstable).

For the equilibrium point $\left(\xi_{2},\psi_{2}\right)=\left(0,\frac{\delta }{\alpha }\right)$ we have:

$J\left(\xi_{2},\psi_{2}\right)\;=\;\left[\begin{array}{ll} 1-\frac{\delta }{\alpha } & 0\\ \beta -\gamma \frac{\delta }{\alpha } & 2\alpha \frac{\delta }{\alpha }-\delta \end{array}\right]\;=\;\left[\begin{array}{ll} 1-\frac{\delta }{\alpha } & 0\\ \beta -\frac{\gamma \delta }{\alpha } & \delta \end{array}\right]\;\Rightarrow |J|=\delta \cdot \left(1-\frac{\delta }{\alpha }\right)$ for the equilibrium point $\left(\xi_{2},\psi_{2}\right)$. The sign of the determinant depends on the term $\left(1-\frac{\delta }{\alpha }\right)$. When δ>α, then |J|<0 (hence one positive and one negative eigenvalue), meaning that the equilibrium point is saddle. This becomes unstable when δ<α. Next, we evaluate the Jacobian matrix in the equilibrium points $\left(\xi_{3,4},\psi_{3,4}\right)$ , by using ξ=1−ψ≥0,

$$\left|J\left(1-\psi ,\psi \right)\right|=\;\left|\begin{array}{ll} -1+\psi & -1+\psi \\ \beta -\;\gamma \psi \; & \left(2\alpha \;+\;\gamma \right)\psi \;-\;\left(\gamma +\delta \right) \end{array}\right|$$

=−(−1+ψ)(β+δ+γ−2(α+γ)ψ)

=−(1−ψ)(2(α+γ)ψ−(β+δ+γ)).

We obtain the trace of the Jacobian matrix, tr(J), to determine the type of stability of equilibrium points $\left(\xi_{3,4},\psi_{3,4}\right)\text{,}$

tr(J)=−1−δ+γ(−1+ψ)+ψ+2αψ=(1+2α+γ)ψ−(1+γ+δ).

We first need to find some critical values for ψ:

• If |J(1−ψ,ψ)|=0, then

(Equation S8) $$\psi_{1}^{*}=\frac{\beta +\gamma +\delta }{2\left(\alpha +\gamma \right)}$$

We know that the term (1−ψ) equals ξ, which represents the bacteria, and hence ξ=1−ψ≥0.

• If tr(J)=0, then

(Equation S9) $$\psi_{2}^{*}=\frac{1+\gamma +\delta }{1+2\alpha +\gamma }$$

From the critical points found in Equations S8 and S9, the stability of the equilibrium points $\left(\xi_{3,4},\psi_{3,4}\right)=\left(1-\psi_{3,4},\psi_{3,4}\right)$ can be classified as follows:

#### Stable node or spiral (represents the chronic phase)

• |*J*| ≥ 0 $\Rightarrow \psi_{1}^{*}\leq \left(\beta +\gamma +\delta \right)/\left(2\left(\alpha +\gamma \right)\right)$
• tr(J) ≤ 0 $\Rightarrow \psi_{2}^{*}\leq \left(1+\gamma +\delta \right)/\left(1+2\alpha +\gamma \right)$

$$\Rightarrow \psi^{*}\leq min\left\{\psi_{1}^{*},\psi_{2}^{*}\right\}$$

##### Saddle equilibrium point

$$\left|J\right|<0\Rightarrow \psi_{1}^{*}>\frac{\beta +\gamma +\delta }{2\left(\alpha +\gamma \right)}\Rightarrow \psi^{*}>\frac{\beta +\gamma +\delta }{2\left(\alpha +\gamma \right)}$$

##### Unstable node or spiral

$$\left|J\right|\;\geq \;0\;\Rightarrow \psi_{1}^{*}\leq \frac{\beta +\gamma +\delta }{2\left(\alpha +\gamma \right)}\Rightarrow \;\psi^{*}\;\leq \psi_{1}^{*}$$

$$tr\left(J\right)\geq \;0\;\Rightarrow \;\psi_{2}^{*}\;\geq \frac{1+\gamma +\delta }{1+2\alpha +\gamma }\;\Rightarrow \psi^{*}\geq \psi_{2}^{*}$$

$$\Rightarrow \;\frac{\beta +\gamma +\delta }{2\left(\alpha +\gamma \right)}\;\geq \psi^{*}\geq \frac{1+\gamma +\delta }{1+2\alpha +\gamma }.$$

However, since from simulation results (Table S1) *c*_*T*_ = 0, we conclude that

$\psi_{1}^{*}=\frac{\beta +\delta }{2\alpha }$ and. $\psi_{2}^{*}=\frac{1+\delta }{1+2\alpha }.$

Substituting α, β, and δ from Equation S4:

$$\psi_{2}^{*}=\frac{1+\frac{\text{Θ}}{r_{b}}}{1+\frac{2r_{t}}{c_{b}}}=\frac{r_{b}+\text{Θ}}{r_{b}+2\text{Z}},\;\text{where Z}=\frac{r_{t}r_{b}}{c_{b}}.$$

Since ξ and ψ are normalized, ξ, ψ ≥ 0 and therefore $0\;\leq \psi_{1}^{*},\psi_{2}^{*}\leq 1.$ Hence $\psi_{2}^{*}\leq 1\;$ resulting in

(Equation S10) Θ≤2Z.

Now, $\psi_{1}^{*}=\frac{\frac{\kappa \;k_{b}\;c_{b}}{r_{b}^{2}}+\frac{\text{Θ}}{r_{b}}}{2\;\frac{r_{t}}{c_{b}}}=\frac{1}{2\text{Z}}\left(\frac{\kappa k_{b}r_{t}}{\text{Z}}+\text{Θ}\right)$.

From Equation S10

$$\psi_{1}^{*}\leq \frac{1}{2\text{Z}}\left(\frac{\kappa k_{b}r_{t}}{\text{Z}}+2\text{Z}\right)=1+\frac{\kappa k_{b}r_{t}}{2\;{\text{Z}}^{2}}>1.$$

As a consequence, $\psi_{1}^{*}$ is rejected and the stability analysis for the equilibrium points $\left(\xi_{3,4},\psi_{3,4}\right)=\left(1-\psi_{3,4},\psi_{3,4}\right)$ can be updated as follows:

• Stable node or spiral (Represents the chronic phase):

$$\psi^{*}\leq \psi_{2}^{*}\Rightarrow \psi^{*}\leq \frac{1+\gamma +\delta }{1+2\alpha +\gamma }\;.$$

• Unstable node or spiral:

$$\psi^{*}\geq \psi_{2}^{*}\Rightarrow \psi^{*}\geq \frac{1+\gamma +\delta }{1+2\alpha +\gamma }\;.$$

#### Simulating the perturbation treatment

To simulate the perturbation strategy, we incorporated, for a perturbation window of 12 hours, the extra term *wB*_*d*_ into the ODE describing T cells (Equation 2), where *B*_*d*_ = 10^8^ cells is the dose of heat-killed *S. aureus* and *w* the parameter of T-cell activation and recruitment by heat-killed treatment. The parameter *w* for the activation and recruitment of T cells via HKSA was assumed to be the same as *k*_*b*_ = 0.001509 [days^−1^], the parameter of T-cell activation and recruitment via live bacteria during initial inoculation (Table S1). This assumption for the value of *w* in the treatment term *wB*_*d*_ was based on the fact that, although live cells produce virulence factors that stimulate the immune system, implying *k*_*b*_ > *w,* heat-killed cells cannot hijack or evade immunity as live *S. aureus* does. The only consequence of the existence of HKSA cells in the host is the stimulation of immune cells. Additionally, HKSA cells are injected when the host is already chronically infected and had already encountered staphylococcal antigens during initial inoculation. Secondary exposure to pathogens always initiates stronger (and quicker) immune response than the initial exposure to the same pathogen, implying *w* > *k*_*b*_. A combination of the two conditions above, led to the assumption *w* = *k*_*b*_, hence the treatment term became *k*_*b*_*B*_*d*_.

The term of heat-killed treatment *k*_*b*_*B*_*d*_ was incorporated into the ODE of T cells, because heat-killed cells, as it happens when administered via conventional vaccines, deliver antigens into the body that enhance the adaptive immunity. On each day (timepoint) of administration of heat-killed treatment, such as day 14 of infection, the treatment term *k*_*b*_*B*_*d*_ was added, and the equations became:

$$\dot{B}\left(t\right)=r_{b}B\left(t\right)\left(1-\frac{B\left(t\right)}{\kappa }\right)-c_{b}T\left(t\right)B\left(t\right)\text{,}$$

(Equation 3) $$\dot{T}\left(t\right)=r_{t}T^{2}\left(t\right)\left(1-\frac{T\left(t\right)}{K_{T}}\right)+k_{b}B\left(t\right)-c_{T}B\left(t\right)T\left(t\right)-\text{Θ}\;T\left(t\right)+k_{b}B_{d}\text{.}$$

Since heat-killed bacteria are not present continuously *in vivo*, the treatment term (that simulates the *in vivo* conditions) was removed after the perturbation window and the equations returned to their initial form as in Equations 1 and 2. For treatment with heat-killed *Streptococcus pyogenes* or varying HK-dose, the HK-dose *B*_*d*_ and value of *k*_*b*_ in the treatment term *k*_*b*_*B*_*d*_ were as stated in the main text.

The estimated day of clearance was defined *in silico* as the first time-point when bacterial numbers were <0.000001.

#### In silico implications to potential clinical implementation

Although administration of inactivated cells as treatment for infections has been used in the past, this kind of therapy is narrowly established. This is due to lacking information regarding the sufficient dose(s) required and day(s) of administration that could resolve *S. aureus* infections successfully. Until now, treatments with inactivated bacteria against *S. aureus* infections have been based only on experimental experience.

Here, we attempt to explain *in silico* why heat- or formalin-killed bacteria treatments used so far have not been successful in yielding bacterial eradication. We base our arguments on a previous clinical trial,^7^ in which at least 19 injections with increased dose of formalin-killed *S. aureus* were administered over a period of 3 months in human patients with *S. aureus* infection (furunculosis).

In the study none of the chronically infected patients was reported to have attained complete bacterial clearance, even though they experienced moderate to strong clinical improvement. The injecting scheme in the study consisted of increasing killed *S. aureus* doses (*B*_*d*_) given in intervals of 3-5 days as follows:

– Suspension I:(0.1,0.2,0.3,0.4,0.5) × 5 × 10^8^
– Suspension II: (0.3, 0.4, 0.5, 0.6, 0.7, 0.8, 0.9, 1) × 10^9^
– Suspension III:(0.5,0.6,0.7,0.8,0.9,1) × 2.5 × 10^9^.

We assumed that the bacterial capacity in humans is 1000 times greater than the bacterial capacity in mice (based on the kidney weight ratio between mice and humans, which is a simple interspecies allometric scaling practice for translating preclinical experiments when designing Phase I clinical studies and scaling drug doses^56^^,^^57^), created the corresponding murine suspensions:

– Suspension IV:(0.1,0.2,0.3,0.4,0.5) × 5 × 10^5^
– Suspension V: (0.3, 0.4, 0.5, 0.6, 0.7, 0.8, 0.9, 1) × 10^6^
– Suspension VI:(0.5,0.6,0.7,0.8,0.9,1) × 2.5 × 10^6^,

and applied them in our mouse model *in silico* every 4 days, starting from day 14, when chronic infection is established in mice (Figure S3A).

### Experimental model and subject details

#### Bacteria

*S. aureus* strain SH1000^54^ was grown to Mid-Log phase in brain heart infusion medium (BHI, Roth, Karlsruhe, Germany) at 37°C with shaking (120 rpm), collected by centrifugation, washed with sterile PBS, and diluted to the required concentration. The number of viable bacteria was determined after serial diluting and plating on BHI-agar.

#### Mice and infection

A previously described chronic renal abscess infection model^10^ has been used in this study. It is known that *S. aureus* strain SH1000 produces renal abscesses after intravenous inoculation.^10^ Pathogen-free, 10 weeks-old C57BL/6 female mice were purchased from Harlan-Winkelmann (Envigo, Netherlands). All animals were provided with food and water *ad libitum*, and housed in groups of up to 5 mice per cage in individually ventilated cages. Statistical power analysis with anticipated incidence of 10% and 90% for control and treated mice, respectively, indicated a sample size of five mice per group. Mice were infected with 5 × 10^7^ CFU of *S. aureus* in 100 μL of PBS via a tail vein and monitored on a daily basis for weight loss and sign of pain or distress. At specified times of infection, mice were sacrificed by CO_2_ asphyxiation and the bacterial load was enumerated in kidney homogenates by plating 10-fold serial dilutions on blood agar plates. “0 CFU” was assigned to samples without detectable bacteria in any of the serial dilutions. Spleens were removed, transformed in a single cell suspension and further processed for FACS and proliferation assays.

In vaccination experiments, infected mice were injected intraperitoneally at day 14 of infection with 10^8^ heat-killed bacteria of *S. aureus* strain SH1000 or *S. pyogenes* strain A20 in 200 μL of PBS that were prepared by heating a bacterial suspension at 60°C for 1 h. At 12 h postchallenge, mice were sacrificed and peritoneal exudate cells (PEC) were isolated from infected mice by lavage of the peritoneal cavity with 2 mL sterile PBS. The lavage fluid was centrifuged, supernatants stored at −20°C for subsequent cytokine analysis, and PEC resuspended in complete RPMI, stained and analyzed by flow cytometry (see below).

Animal experiments were performed in strict accordance with the German regulations of the Society for Laboratory Animal Science (GV-SOLAS) and the European Health Law of the Federation of Laboratory Animal Science Associations (FELASA). All experiments were approved by the ethical board Niedersächsisches Landesamt für Verbraucherschutz und Lebensmittelsicherheit, Oldenburg, Germany (LAVES; permit N. 18/2798).

#### Flow cytometry analysis

Cells were incubated with purified rat anti-mouse CD16/CD32 (BD Biosciences) for 5 min to block Fc receptors and then stained with antibodies against CD11b (BioLegend), Ly6C (BioLegend), Ly6G (Miltenyi Biotec), F4/80 (BD Biosciences) for 20 min at 4°C. Labeled cells were measured by flow cytometry using a BDTM LSR II flow cytometer (BD Biosciences) and analyzed by FlowJo software.

#### Proliferation assay

Spleen cells were seeded into 96-well flat-bottom plates at 5 ×10^5^ cells/well in 100 μL of complete RPMI medium and stimulated with 2 μg/mL of anti-CD3/anti-CD28 antibodies (Sigma-Aldrich) at 37°C and 5% CO_2_. After 3 days of incubation, the cells were pulsed with 1 μCi ^3^H-thymidine (Amersham) and harvested 18 h later on Filtermats A (Wallac) using a cell harvester (Inotech). The amount of ^3^H-thymidine incorporation was measured in a gamma scintillation counter (Wallac 1450; MicroTrilux).

### Quantification and statistical analysis

All data were analyzed with GraphPad Prism 7.0 Software. Comparisons between several groups were made using a parametric ANOVA test with Tukey’s multiple comparisons test. Comparison between two groups was performed using a Student’s t-test. p values <0.05 were considered significant.

## Data Availability

• Raw experimental data have been deposited at Mendeley and are publicly available as of the date of publication. The DOI is listed in the key resources table. • The MATLAB codes used for simulations are available from the corresponding authors upon reasonable request. • Any additional information required to reanalyze the data reported in this paper is available from the lead contact upon request.
