# Supplementary material for: In silico predicted therapy against chronic Staphylococcus aureus infection leads to bacterial clearance in vivo
Source: iScience. 2022 Nov 8;25(12):105522. doi: 10.1016/j.isci.2022.105522 (PMC9700272; doi:10.1016/j.isci.2022.105522)
Supplement: Document S1. Figures S1–S5 and Table S1 [file mmc1.pdf]

**iScience, Volume *25***

## Supplemental information

# *In silico* predicted therapy against chronic *Staphylococcus aureus* infection leads to bacterial clearance *in vivo*

**Lito A. Papaxenopoulou, Gang Zhao, Sahamoddin Khailaie, Konstantinos Katsoulis-Dimitriou, Ingo Schmitz, Eva Medina, Haralampos Hatzikirou, and Michael Meyer-Hermann**

## Supplemental figures

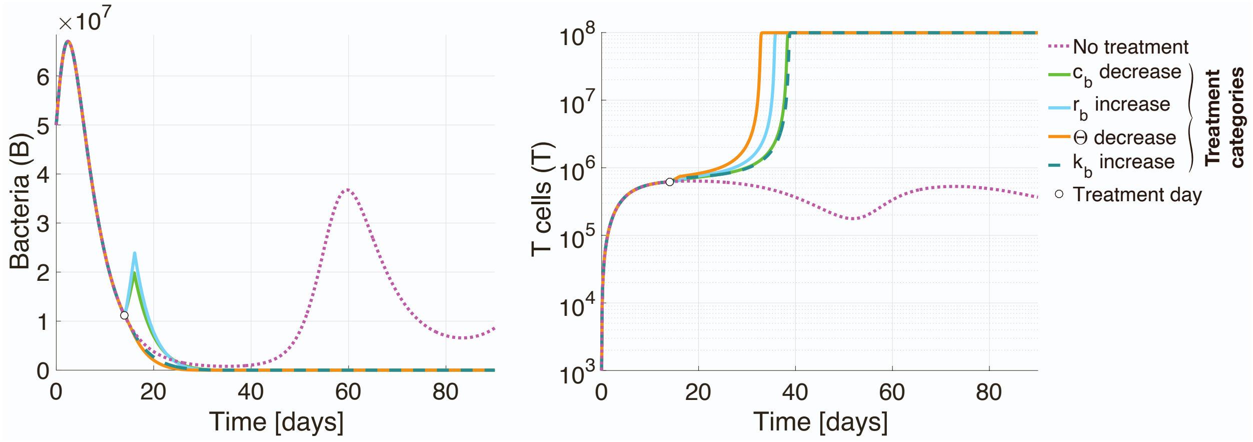


**Figure S1. Perturbation strategies suggest eradication of *S. aureus in silico*, related to Figure 2.** Progression of *S. aureus* infection without any perturbation treatment results in a mathematically-characterized stable state and in a clinically-characterized persistent (chronic) *S. aureus* infection. When the chronic infection system is perturbed with treatments that (i) decrease host aggression against bacteria ($c_b$), for example via immunosuppressive drugs, (ii) increase bacterial growth ($r_b$), (iii) reduce MDSC-mediated immunosuppression ($\Theta$), for instance via gemcitabine chemotherapeutic drug, or (iv) boosting adaptive immunity of the host ($k_b$), such as via administration of heat-killed bacterial antigens, *S. aureus* is eradicated. The plotted bacterial and T-cell dynamics in time are the numerical solutions of the ODE system (Eqs. (1)-(2)). Treatments were applied *in silico* at day 14 of infection (white bullet), namely when infection was already chronic, by decreasing or increasing the fitted value of the parameter of interest (table S1) for a perturbation window of 12 hours up to two days.

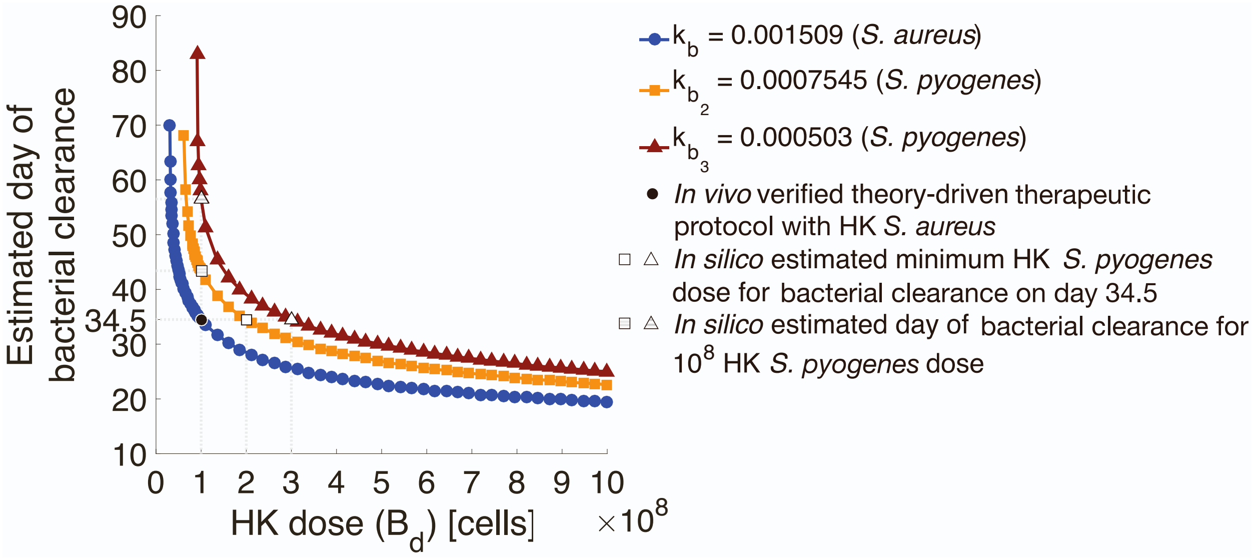


**Figure S2. Time of bacterial clearance for varying HK-doses and three values of T-cell stimulatory parameter $k_b$ *in silico*, related to Figure 4.** Stimulation of T cells by HK *S. aureus* cells at day 14 happens with rate $k_b = 0.001509$ as defined during the fitting process (table S1). Since primary exposure to *S. aureus* already occurs upon inoculation at day 0, HK *Streptococcus pyogenes* at day 14 of infection stimulates the immune system at a weaker and slower rate compared to HK *S. aureus*, assumed as $k_{b2} = k_b/2 = 0.0007545$ or $k_{b3} = k_b/3 = 0.000503$. Simulation of HK treatments was done by adding the term $k_b B_d$ to the T-cell ODE at day 14 of infection for a perturbation window of half day (STAR Methods), where $k_b$ the immunostimulatory parameter varying for three different values ($k_b$, $k_b/2$, $k_b/3$) and $B_d$ the HK-doses varying in the range $[4\times10^7, 10^9]$. The estimated day of clearance was defined as the first time-point when bacterial numbers <0.000001.

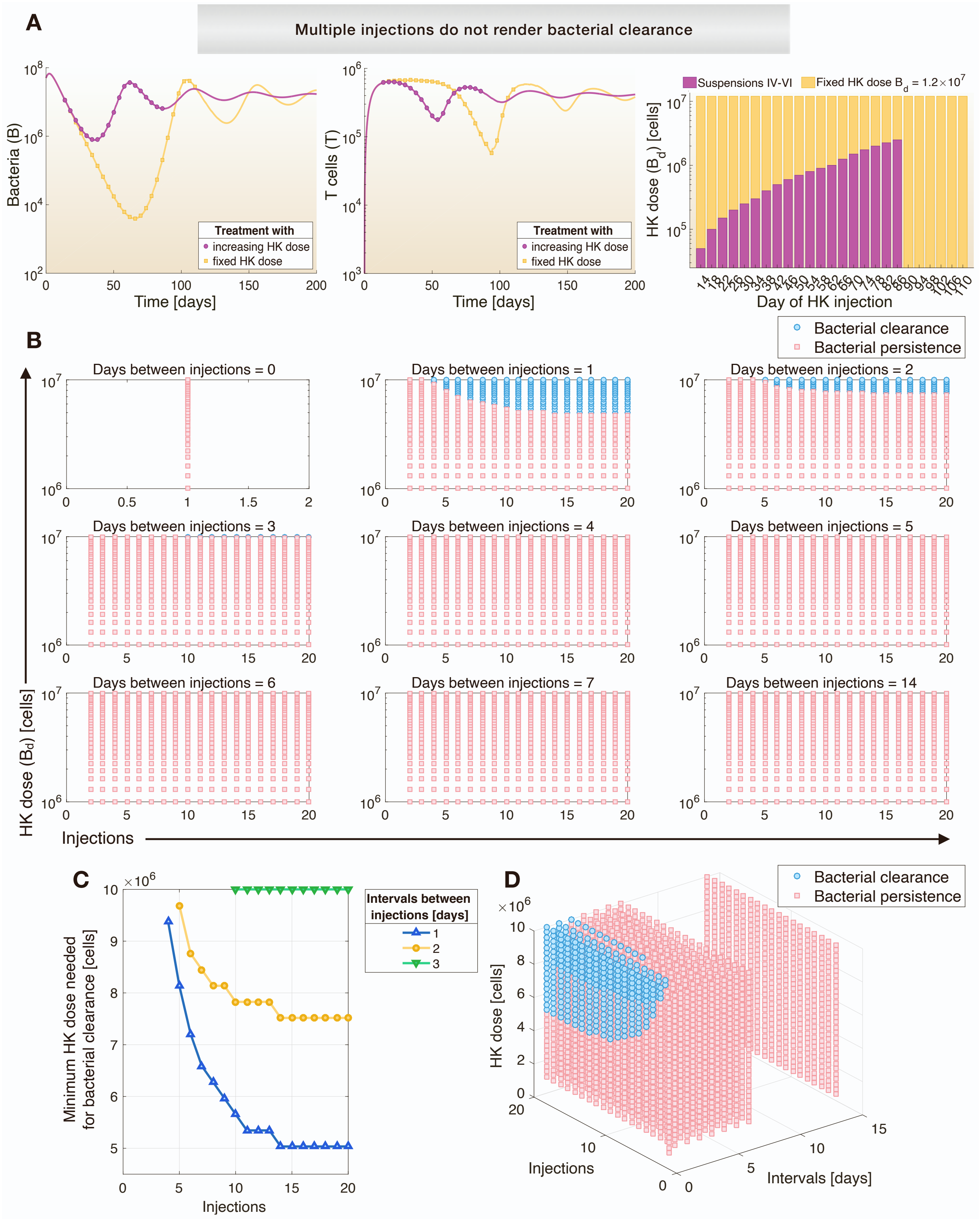


**Figure S3. Simulations explain why previous treatments with inactivated *S. aureus* in humans were unsuccessful and suggest therapeutic treatment protocols, related to STAR Methods and**

**Figure 2.** At each injection day starting from day 14 of infection (when infection is chronic), treatment was incorporated into the T-cell ODE (Eq. (2)) as $k_b B_d$, where $k_b$ as defined in table S1 and $B_d$ the varying, administered HKSA-dose. (**A**) Repeated administration of inactivated *S. aureus* cannot render clearance *in silico*. 19 HKSA-injections with increasing dose in an analogous way to as administered in humans (suspensions IV-VI, purple) or 25 injections with high fixed dose (yellow) were administered *in silico* every four days, starting from day 14. Chronic *S. aureus* infection persists. (**B-D**) *In silico* predictions for bacterial clearance plotted for 1 up to 20 HKSA-injections, HKSA-doses ranging from $10^6$ to $10^7$, and time intervals between HKSA-injections from 0-7 days, or 2 weeks. Time interval of 0 days means that only one HKSA-injection is administered. (**B**) Possibility of bacterial clearance declines when time intervals between HK-injections increase, even if many injections with high HKSA-dose are administered. For doses of $10^6$ to $10^7$ inactivated *S. aureus,* only treatment protocols involving HK injections with time intervals from 1-3 days could resolve the infection (*blue*), with the maximum time interval of 3 days requiring a minimum dose of $10^7$ inactivated *S. aureus* to be effective. Each blue circle represents a unique, therapeutic treatment protocol and shows that bacterial clearance is achieved only when time intervals between injections are 1-3 days with minimum HKSA-dose needed for bacterial clearance as in (**C**). (**D**) Interconnection between HKSA-doses $10^6$ to $10^7$, HKSA-injections and time intervals between injections in three-dimensional plot.

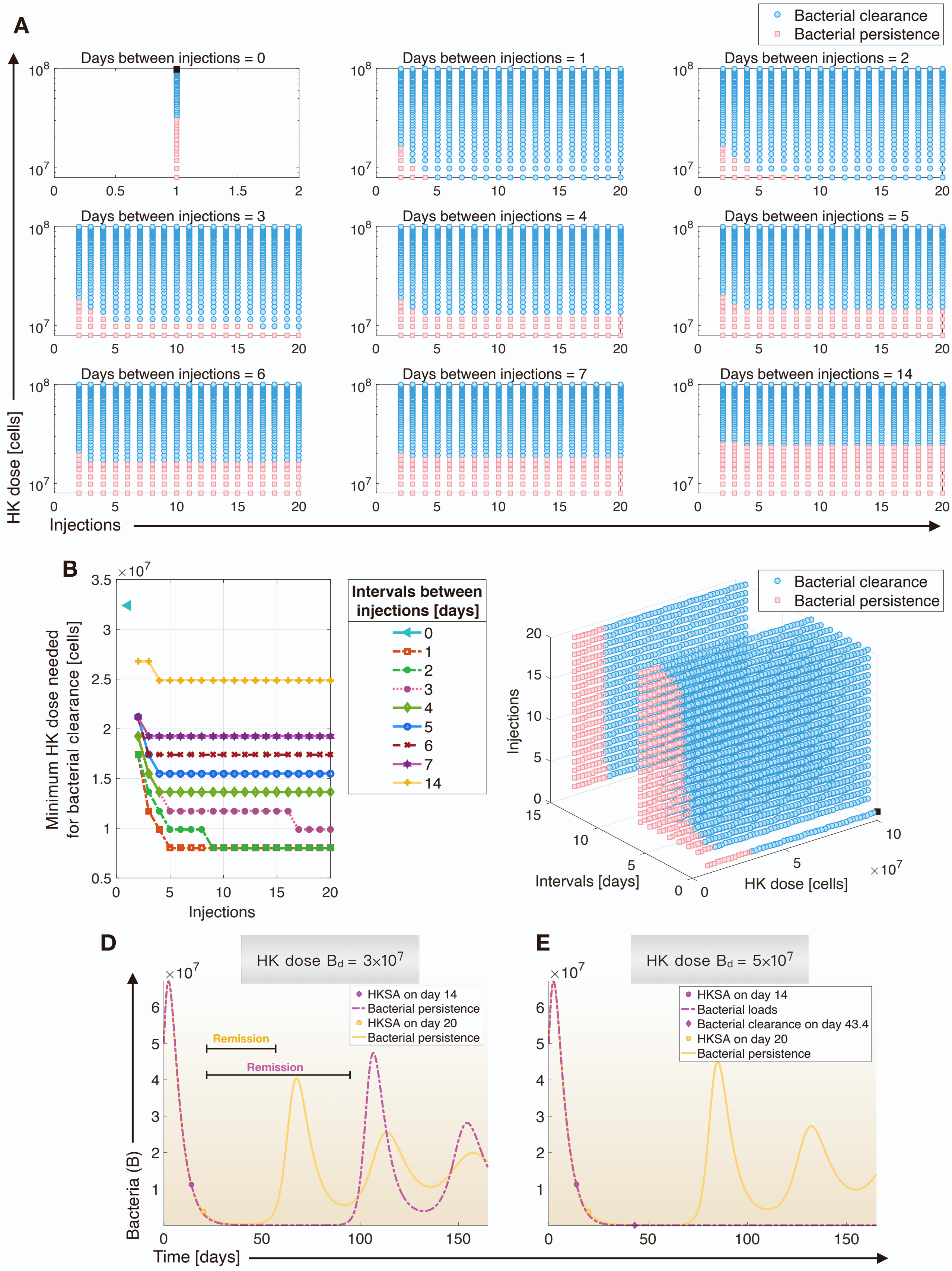

A
Bacterial clearance
Bacterial persistence
Days between injections = 0
Days between injections = 1
Days between injections = 2
Days between injections = 3
Days between injections = 4
Days between injections = 5
Days between injections = 6
Days between injections = 7
Days between injections = 14
HK dose [cells]
Injections
B
Minimum HK dose needed for bacterial clearance [cells]
Intervals between injections [days]
0
1
2
3
4
5
6
7
14
Injections
Bacterial clearance
Bacterial persistence
Injections
Intervals [days]
HK dose [cells]
D
HK dose Bd = 3x10^7
HKSA on day 14
Bacterial persistence
HKSA on day 20
Bacterial persistence
Remission
Remission
Bacteria (B)
Time [days]
E
HK dose Bd = 5x10^7
HKSA on day 14
Bacterial loads
Bacterial clearance on day 43.4
HKSA on day 20
Bacterial persistence

**Figure S4.** ***In silico*** **correlation between HKSA-doses from $8\times10^6$ to $10^8$, number of HKSA-injections and time intervals between injections for therapeutic treatment protocols or remission, related to Figure 2.** (**A-C**) Same as in Figures S3B-S3D for HKSA-doses ranging from $8\times10^6$ to $10^8$. Each blue circle represents a unique, therapeutic treatment protocol, for which the minimum dose required for successful treatment is indicated in (**B**). Black square in (**A**) and (**C**) represents the treatment protocol of $10^8$ HKSA in a single injection that was tested experimentally in this study and eliminated *S. aureus* in all hosts *in vivo*. (**D-E**) Influence of administration time of HKSA-injection on the chronic infection. The HKSA-treatment *in silico* was added as term $k_b B_d$ to the T-cell ODE (Eq. (2)) at day 14 or 20 for a 12-hour perturbation window, where $k_b$ as in table S1 and $B_d$ the HKSA-dose. Critical HKSA-dose required for bacterial clearance when administered at day 14 (early chronic infection) was estimated to be $4\times10^7$ (Figure 2C). Although lower HKSA-dose (**D**) for example $B_d = 3\times10^7$ HKSA cannot confer bacterial elimination, it provides remission. Higher HKSA-dose (**E**), such as $B_d = 5\times10^7$, confers bacterial clearance when administered at early timepoints of chronic infection but fails to render bacterial clearance when administered later, although it provides remission temporarily.

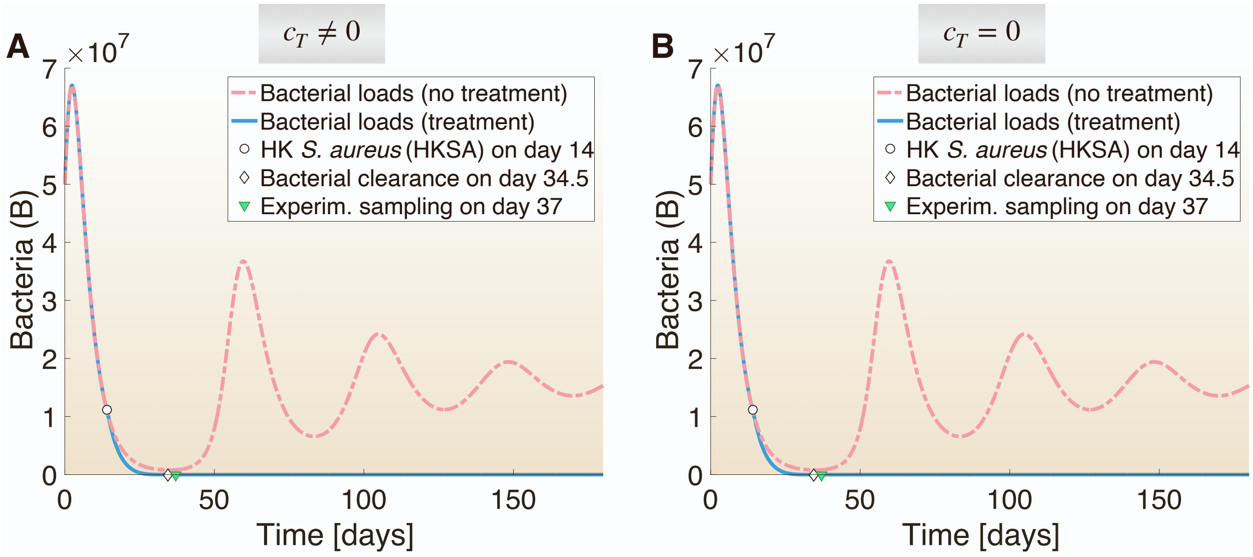


**Figure S5. Absence of the local immunosuppression effect does not influence the model's therapeutic predictions, related to STAR Methods.** Including or excluding the local immunosuppression was done by setting the corresponding parameter $c_T$ equal to either **(A)** $2.2204\times10^{-16}$ (estimated by the first fitting process) or **(B)** zero. Simulation of the HK treatment was done by adding the term $k_b B_d$ to the T-cell ODE at day 14 of infection for a perturbation window of half day (STAR Methods), where $k_b$ = 0.001509 days$^{-1}$ the immunostimulatory parameter as defined during the fitting process (table S1) and $B_d = 10^8$ the dose of HKSA. The estimated day of clearance was defined as the first time-point when bacterial numbers <0.000001. The day of clearance was predicted to be the day 34.5 post-infection, irrespectively of the given value of parameter $c_T$ (namely either $2.2204\times10^{-16}$ or 0).

## Supplemental table

**Table S1. Model parameter values as used for model analysis, related to Figures 1-2 and STAR Methods.**

| Parameter | Description | Value, [Confidence Interval]* | Unit |
|---|---|---|---|
| $r_b$ | Bacterial growth rate (in the presence of innate immunity) | 0.477 | days$^{-1}$ |
| $\kappa$ | Carrying capacity of bacteria | $1.132 \times 10^{8}$ | cells |
| $c_b$ | Rate of bacterial containment by T cells (per cell) | $9.937 \times 10^{-7}$, $[8.65\times10^{-7}, 1.25\times10^{-6}]$ | days$^{-1}$ |
| $r_t$ | T-cell proliferation rate (per cell) | $2.0955 \times 10^{-7}$, $[1.04\times10^{-7}, 2.94\times10^{-7}]$ | days$^{-1}$ |
| $k_b$ | T-cell activation and recruitment rate | 0.001509, [0.0011, 0.0017] | days$^{-1}$ |
| $c_T$ | Local T-cell suppression rate (per cell) | 0 | days$^{-1}$ |
| $\Theta$ | Systemic MDSC-mediated suppression rate on T cells | 0.14393, [0.072, 0.18] | days$^{-1}$ |
| $K_T$ | Carrying capacity of T cells | $10^{8}$ | cells |

* In square brackets is the 95% confidence interval of the parameters as derived by a Markov Chain Monte Carlo version of Differential Evolution algorithm [S1].

## SUPPLEMENTAL REFERENCES

1. Braak, C.J.F.T. (2006). A Markov Chain Monte Carlo version of the genetic algorithm differential evolution: easy Bayesian computing for real parameter spaces. *Stat. Comput.* **16**, 239-249. 10.1007/s11222-006-8769-1.
